# A synbiotic mixture of selected oligosaccharides and bifidobacteria assists murine gut microbiota restoration following antibiotic challenge

**DOI:** 10.1186/s40168-023-01595-x

**Published:** 2023-08-02

**Authors:** Emily C. Hoedt, Cara M. Hueston, Nora Cash, Roger S. Bongers, Jonathan M. Keane, Kees van Limpt, Kaouther Ben Amor, Jan Knol, John MacSharry, Douwe van Sinderen

**Affiliations:** 1 APC Microbiome Ireland, University College Cork, Western Road, Cork, Ireland; 2 Current address: NHMRC CRE in Digestive Health, HMRI, Newcastle, NSW Australia; 3 Danone Nutricia Research, Utrecht, The Netherlands; 4 Laboratory of Microbiology, Wageningen University, Wageningen, The Netherlands; 5 School of Microbiology, University College Cork, Western Road, Cork, Ireland; 6 School of Medicine, University College Cork, Cork, Ireland

## Abstract

**Background:**

Typically, animal models studying gastrointestinal microbiotas compromised in early life have employed either germ-free animals or mice treated with a cocktail of antibiotics. Such studies intend to mimic scenarios of infants born by caesarean section and/or subjected to antibiotic treatment. However, the antibiotics used in these studies are rarely prescribed to infants. Therefore, an early life model was developed in which the murine gastrointestinal microbiota was severely disrupted by clindamycin treatment.

**Results:**

In this mouse model, we investigated the extent supplementation with a synbiotic mixture of prebiotics, being scGOS/lcFOS with the human milk oligosaccharide 2’-Fucosyllactose (2’-FL), in combination with or without single strain or mix of “infant type” bifidobacteria, can rescue an antibiotic-compromised microbiota. Shotgun metagenomic sequencing showed that the microbiota was severely disrupted by the clindamycin challenge. No recovery was observed 3 weeks post-challenge in the scGOS/lcFOS/2’FL group, while the group that received the synbiotic treatment of scGOS/lcFOS/2’-FL with *Bifidobacterium breve* NRBB01 showed partial recovery. Strikingly in the scGOS/lcFOS/2’-FL group receiving the mixture of bifidobacteria resulted in a recovery of the microbiota disruption. Histological analyses showed that the clindamycin-treated animals at the end of the experiment still suffered from mild oedema and villi/colonic crypt irregularities which was ameliorated by the synbiotic intervention.

**Conclusion:**

Our study demonstrates that supplementation of synbiotic mixture of scGOS/lcFOS/2’-FL in combination with a specific mix of infant-type bifidobacterial strains is able to partially revive an antibiotic-perturbed gastrointestinal microbiota.

Video Abstract

**Supplementary Information:**

The online version contains supplementary material available at 10.1186/s40168-023-01595-x.

## Introduction

During the first 1000 days following birth, the developing microbiota plays a pivotal role in orchestrating long-term health [1–4]. Gastrointestinal tract (GIT) microbiota maturation during this vulnerable period is driven by an appropriate succession of microbes that confer resilience to perturbations [3, 4]. Different environmental factors such as C-section, preterm birth and antibiotic administration can drastically alter this microbial consortium and may confer long-term health issues [5–7]. Ensuring the appropriate compositional balance of the microbiota is considered an effective measure in maintaining GI health. Bifidobacteria are among the keystone GIT colonizers during early infancy and exert positive health benefits on their host. Following birth, bifidobacteria typically become the dominant species in the colon of breast-fed infants, with “infant type” bifidobacteria specifically abundant in this ecological niche [1, 8, 9]. Human milk oligosaccharides (HMOs) act as important carbohydrate substrates creating this ecological niche [1, 10, 11]. However, there is much we still do not understand about specific host and microbiota interaction capabilities of bifidobacteria. As a result, there is ever-increasing interest from a scientific and applied perspective to study bifidobacteria, and their interactions within the microbiota and their host.

Antibiotic administration is common in the first 6 months after birth [12]. A study in the USA found that 75% of infants had been treated with an antibiotic within the first 48 h following birth [13]. Antibiotic treatment, C-section, preterm birth, and diet drastically alter the GI microbiota (including the prevalence and abundance of bifidobacteria [14, 15]). Further, GI microbiota disruption has been linked to developmental alterations, including allergic asthma, impaired growth, obesity, and metabolic disease [5, 16–19]. Clindamycin is a broad-spectrum lincosamide antibiotic, commonly used to treat infections by anaerobic pathogens in both infants and adults, this includes *Staphylococcus aureus* [20] and *Bacteroides fragilis* [21, 22]. In the case of breast-feeding mothers treated with clindamycin, substantial levels of the antibiotic are present in breast milk [23, 24]. It is still unclear whether the use of prebiotics, probiotics or synbiotics confer microbial resilience against the negative impact of antibiotic exposure on the infant GI microbiota. Synbiotics [25], employ both prebiotics (e.g., HMO 2′-Fucosyllactose (2′-FL), galacto-oligosaccharides (scGOS) and fructo-oligosaccharides (FOS)), and probiotics (e.g., *Bifidobacterium* spp.). Given the huge cost associated with antibiotic treatment of disease and adverse effects on health and development, understanding mechanisms which orchestrate potential microbiota resilience and recovery is crucial.

Several studies examine the restorative and beneficial properties of probiotics following disruption of the GI microbiota [26–29]. While there is reported efficacy of single probiotic strains, such as reduction of intestinal inflammation with *B. bifidum* JCM1254 [27], there is some evidence to suggest that a multi-strain probiotic or synbiotic treatment can restore a compromised microbiota [30, 31]. In fact Suez et al. [26] reported based on animal model and human studies that post-multi-antibiotic administration of a multi-strain probiotic based on infant-derived bacteria comes at a trade-off of delayed autochthonous microbiota re-establishment (at least 5 months following the probiotic treatment [26]). None of these studies assessed the impact of synbiotics and thus, the aim of our work was to assess a synbiotic mixture of prebiotics and bifidobacteria to rescue a compromised or perturbed microbiota in early life. Here, we use conventional mice in which the microbiota had been compromised by the commonly applied infant antibiotic clindamycin and aimed to examine if and how synbiotic treatment may be able to support host GI microbiota recovery.

## Materials and methods

### Probiotic strains

The mixture of strains was selected after extensive, in-house screening of various individual *Bifidobacterium* strains and mixtures thereof based on several criteria. First of all, only strains were selected of the “infant” type, with excellent safety profiles and gastrointestinal survival, and their ability to grow on GOS/FOS/HMO’s. The mixture of the two *B. breve* strains (NRBB01 and NRBB57), who are phylogenetically and phenotypically distinct from each other within the *B. breve* species was selected based on previous work [32], as was *B. bifidum* CNCM-I 4319 [33]. It was this mixture that showed the strongest synergy based on growth when cultivated on 2’-FL and scGOS/lcFOS/2’-FL. This synergy in growth appeared to be based on cross-feeding activities, resulting in enhanced growth as also indicated by increased levels of (bifido)bacterial metabolites, like acetate, L-lactate, and 1,2-propanediol formed as for the individual strains. Not all “infant” type bifidobacterial strains show these synergies, for instance when *B. infantis* was grown in combination with a *B. breve* strain on 2’-FL even an antagonism was seen on growth of *B. infantis*.

Strains selected for this study (Table S1) were *Bifidobacterium breve* NRBB01*,* *B. breve* NRBB57 and *B. bifidum* CNCM I-4319. Introduction of plasmids into bifidobacterial strains by electroporation was performed as described previously [34, 35] with plasmids each containing a particular selective marker: pSKEM (erythromycin resistance in NRBB01), pPKCM (chloramphenicol resistance to NRBB57) or pDM1 (spectinomycin resistance to CNCM I-4319) [36, 37]. Transformants were incubated anaerobically at 37 °C until an optical density (OD_600nm_) of ~ 1 was reached. Bacterial cells were then harvested by centrifugation (4052 × *g* for 20 min) and washed twice with pre-warmed (37 °C) PBS (37 mM NaCl, 2.7 mM KCl, 10 mM Na_2_HPO_4_, and 1.8 mM KH_2_PO_4_). After the final wash, cells were gently resuspended in 20% glycerol: 80% PBS solution (vol/vol) to obtain a stock with CFU/mL of ~ 1 × 10^9^. 1 mL aliquots of this cell suspension were transferred to sterile 2 mL screw cap tubes and frozen at − 80 °C. Stocks were assessed for viable count and confirmed to have remained at ~ 1 × 10^9^ CFU/mL. The bifidobacterial mix was made as described above with the exception that equal volumes of each strain were mixed before glycerol stocks were aliquoted. This mix was plated to confirm the CFU equalled ~ 1 × 10^9^ CFU/mL.

### Animals

Three-week old female C57BL/6 mice were obtained from Envigo, UK. Mice were randomly housed in individually ventilated cages (each holding 5 mice) with enrichment materials (cardboard tubes, nesting material, chew sticks), and allowed to acclimatise for 7 days prior to the start of experimentation. Animals were fed when first received and ad libitum throughout the whole study with (AIN93G diet [38] or the AIN93G + scGOS/lcFOS/2’-FL) enriched diet, manufactured by SSNIFF (Table S2). The mouse holding room was maintained at 21 ± 1 °C with humidity of 55 ± 10% and a 12-h/12-h light/dark cycle. All efforts were made throughout the experiments to minimize animal suffering and to reduce the number of animals used.

### Optimization trial

**Fig. 1:**
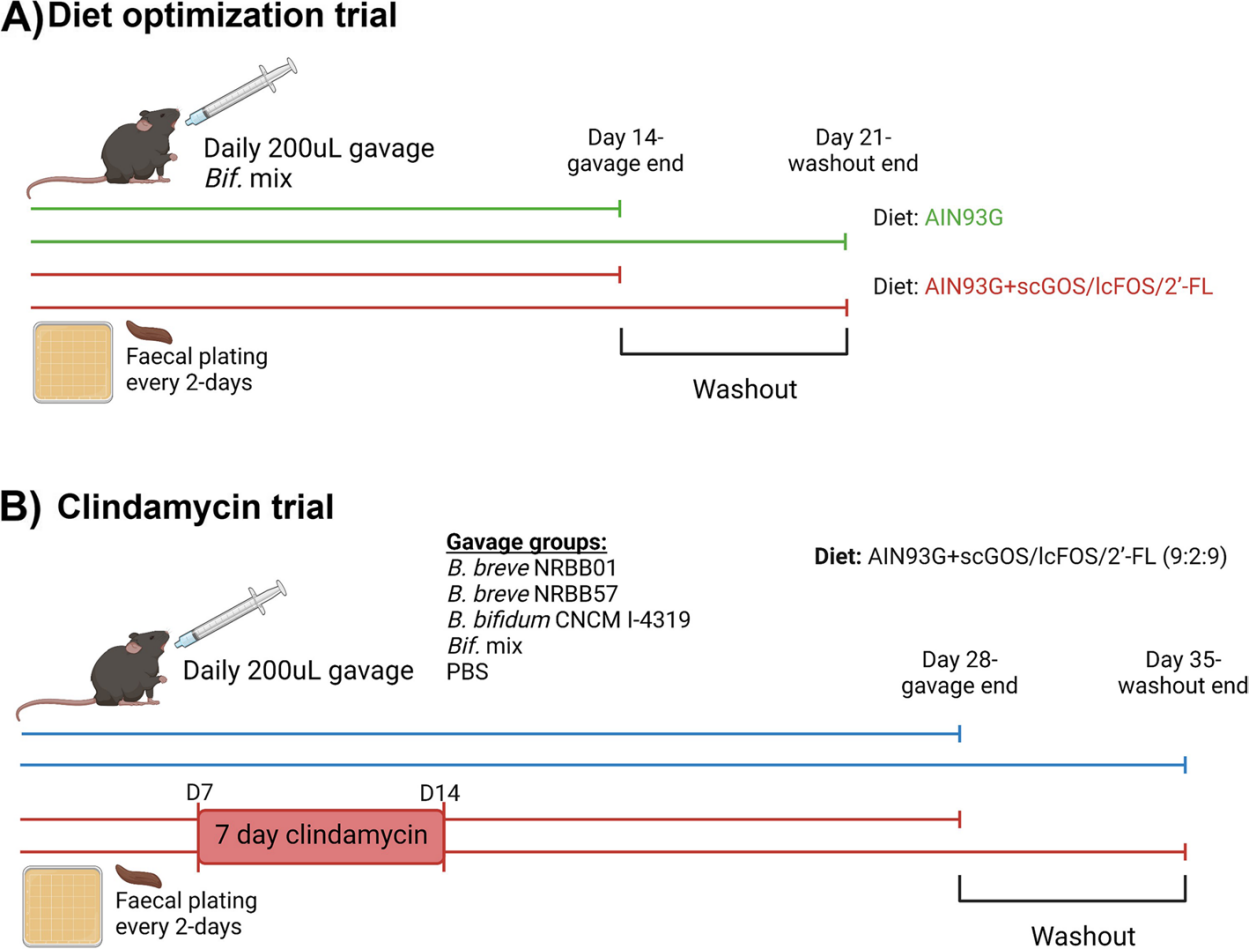
**A** Diet Trial outline, animals (*n* = 7/group) received daily the bifidobacterial strain mix (*B. breve* NRBB01, *B. breve* NRBB57 and *B. bifidum* CNCM I-4319) by oral gavage for 14 days while being fed either the AIN93G control diet (green) or the prebiotic diet (AIN93G + scGOS/scFOS/2’-FL; red) before being culled. A washout period of 7 days was used in replicate groups before being culled to determine bifidobacterial strain colonization. **B** Clindamycin Trial outline, Animals were gavaged daily for 28 days while being fed the prebiotic diet. Five groups of animals (*n* = 10/group) were used for this purpose (PBS gavage control, *B. breve* NRBB01, *B. breve* NRBB57, *B. bifidum* CNCM I-4319 or *Bif.* mix), of which half of the animals received antibiotic treatment. From day 7 to day 14 animals in the antibiotic challenge arm (red) were given 250 mg L^−1^ of clindamycin as part of the water supply. A cohort of animals were culled 2 weeks post-antibiotic challenge. A second cohort (same five groups, n = 10/group) was given a further 7 days as a gavage washout before being culled to determine bifidobacterial strain colonization. Image created with BioRender

Mice were fed the AIN93G control diet [38] or the AIN93G:scGOS/lcFOS/2’-FL (9:1:2) prebiotic diet (2.4% w/w carbohydrate substitution; Table S2). In this trial (*n* = 7/group replicated twice (each group consisted of 3 cages (total of 14 mice) in which one cage housed 4 mice while the other cages housed 5 mice); Fig. 1A), we aimed to optimize a feed enriched with a specific prebiotic mixture (scFOS/lcFOS and 2’-FL) to support growth of the supplemented probiotic strains *B. breve* NRBB01*, B. breve* NRBB57 and *B. bifidum* CNCM I-4319 in order to enhance their level of colonisation. Animals received a daily oral gavage, of 200 µL of the 1 × 10^9^ CFU stock mixture of three bifidobacterial strains, NRBB01*,* NRBB57 and CNCM I-4319 for 14 days. Replicate groups of animals were additionally left for a final 7 days of bifidobacterial “washout” before being culled (*n* = 7/group). Mice were fed the AIN93G diet [38] or AIN93G:scGOS/lcFOS/2’-FL (9:1:2; Table S2) for the duration of the trial. To determine colonization levels of the autochthonous and administered bifidobacteria, fecal samples were collected every 2 days into 1 mL PBS containing 0.05% cysteine. Samples were weighed, resuspended, and diluted fourfold and 5 µL quantities of the dilutions were spot plated onto selective reinforced clostridium agar with mupirocin (200 µg/mL; RCA-MUP) with appropriate antibiotic selection (Table S1) for each respective strain. Plates were incubated anaerobically at 37 °C after which bifidobacterial viable counts were performed (expressed in CFU/g). DNA from fecal and colony samples were used as a template for standard PCR using strain-specific primers for strain validation during the trial (Table S3). Mice were culled at the end of the trial for GIT tissue (small intestine, cecum and colon) and content (small intestine and caecum) collection which were stored at − 80 °C for subsequent analysis (qPCR and HPLC).

### Clindamycin trial

The clindamycin trial (Fig. 1B) involved daily gavage with 200 µL of 1 × 10^9^ CFU of an individual strain or the mixture of bifidobacterial strains. Mice were fed a modified AIN93G supplemented with scGOS/lcFOS/2’-FL diet with an increased amount of 2’-FL (4% w/w carbohydrate substitution with scGOS/lcFOS/2’-FL (9:2:9), see Supplementary Table S2). Five groups (*n* = 10/group; 5 animals/cage) of animals were used for this purpose (PBS gavage vehicle control, *B. breve* NRBB01, *B. breve* NRBB57, *B. bifidum* CNCM I-4319 or the Mix of the 3 strains). Five animals from each group (of 10 animals) received antibiotic treatment. Mice received a daily administration, by oral gavage, with respective bifidobacteria stocks for 28 days, and on day 7 the antibiotic treatment (clindamycin) was administered in the water supply (250 mg/L; [39]). Faecal samples were collected as above every 2 days to assess total and strain-specific *Bifidobacterium* levels. At the end of the trial animals were culled for the collection of colon, caecum, and small intestine content/tissue for analysis of CFU/g of content and stool, qPCR of host factors from tissue, HPLC of GI content and tissue for histological purposes. The experiment was repeated as described above with the only modification being that animals were left for an additional 7 days to monitor bifidobacterial “washout” before being culled at day 35 after which the same samples were collected for analysis as outlined above.

### Collection of samples for HPLC metabolite analysis

Ileum and cecum contents were collected into pre-weighed Eppendorf tubes with 300 µL high-performance liquid chromatography (HPLC) grade H_2_O (Sigma-Aldrich, Germany). Weights of contents were calculated and the tubes vortexed until contents were completely resuspended. Resuspensions were then centrifuged at 13,000 rpm for 20 min at room temperature and supernatant transferred to High Recovery Vials (Agilent Technologies, Ireland). Carbohydrates and metabolites (glucose, lactose, lactate, acetic acid, formic acid, propionate and ethanol) were determined by an Agilent 1200 HPLC system with a refractive index detector (Agilent Technologies, Ireland). A REZEX 8µ 8%H, Organic Acid Column 300 × 7.8 mM (Phenomenex, USA) is used with 0.01N H_2_SO_4_ as the elution fluid, at a flow rate of 0.6 mL/min. The temperature of the column was maintained at 65 °C. Substrate and end-product peaks were identified by comparison of their retention times with standards of pure compounds and known concentrations.

### GIT tissue collection and preparation for histology

Approximately 1–1.5 cm of ileum and terminal colon segments from each mouse were collected post-mortem from the Clindamycin Trial at day 29 and 35. All tissues were fixed for 2.5 h in methacarn (methanol-Carney) fixative (60% (v/v) dry methanol, 30% (v/v) chloroform, 10% (v/v) glacial acetic acid [40, 41]). The tissues were automatically processed by a Leica Tissue Processor TP1020 Histokinette (Leica Biosystems, UK). Tissue samples were then embedded in paraffin blocks using a Sakura Tissue-Tek TEC5CMA-1 Tissue Embedding Station (Sakura Finetek, USA). Tissue segments were oriented for longitudinal and transversal sectioning. Five micro meters of tissue sections were made using a Leica RM2135 Rotary Microtome (Leica Biosystems, UK).

### Histological staining and scoring

Paraffin-embedded sections were deparaffinised using Clear-Advantage Xylene Substitute (Polyscience, USA) and rehydrated through an ethanol gradient. Haematoxylin and Eosin (H&E) staining for epithelium integrity was performed immediately after deparaffinisation by a Haematoxylin and Eosin Stain Kit (Vector Laboratories, USA) following the manufacturer’s instructions. In addition, replicate slide sections were stained with Alcian Blue/Periodic acid–Schiff (PAS) staining following the manufacturer’s instructions (Polyscience, USA) to visualise mucosal variations. Slides were then imaged using an Olympus BX51 upright microscope and sections were scored as described in previous work [42–45]. All scoring was blinded to experimental conditions with reference to the group only fed the prebiotic diet (no bifidobacterial gavage) to evaluate gross changes compared to control samples in (1) gland number (colon) or villus structure (small intestine), (2) oedema and at the cellular level with (3) goblet cell number, (4) mucus thickness, and (5) acid/neutral mucin distribution from crypts to lumen.

### RNA extraction from tissue

Total RNA was isolated from approximately 1–1.5 cm of mid colon harvested at respective end points and stored in RNAlater at − 80 ℃. Following thawing and removal of the RNAlater, tissue was lysed by bead beating in lysis buffer, followed by RNA purification using the GenElute™ Mammalian Total RNA miniprep kit (Sigma-Aldrich, Germany) as per manufacturer’s protocol instructions. RNA was quantified using a Nanodrop and 1 μg was then converted to cDNA with the ReadyScript™ cDNA Synthesis Mix (Sigma-Aldrich) as per manufacturer’s instructions. RT-qPCR was performed, with primers targeting specific genes of interest (Table S3). Briefly, 1 μL of cDNA was added to 10 μL of power KiCqStart SYBR Green qPCR ReadyMix (Sigma-Aldrich) with 0.5 μL of each forward and reverse primer pair (5 pmol/μL) and made up to a final volume of 20 μL with molecular grade H_2_O. qPCR was performed using the LightCycler 480 System (Roche Diagnostics International AG, Switzerland) following conditions: 95 °C for 5 min, followed by 45 cycles at 95 °C for 10 s, and annealing/extension at 60 °C for 1 min. Relative expression was calculated against β-actin using the 2-^ΔΔCT^ method.

### DNA extraction, sequencing, and analysis

Faecal gDNA was extracted using QIAmp Fast DNA Stool Mini Kit (Qiagen) following manufacturer’s instructions, with one modification being a 3-min bead beating step incorporated at the start of the protocol. Paired-end metagenomic shotgun sequencing (MGS) was completed at the Australian Centre of Ecogenomics (ACE) and consisted of QC, indexing, quantification, normalisation, pooling, and sequencing on a 2 × 300 bp V3 MiSeq. Resulting data was demultiplexed and uploaded to One Codex (One Codex, USA [46]) for taxonomic assignment (https://www.onecodex.com/). The One Codex database consists of ~ 115,000 complete microbial genomes, assembled from both of public and private sources. The mouse genome was included in the initial process to screen out host reads. Samples were annotated against the One Codex database through three sequential steps: (1) *k*-mer based classification (*k* = 31); (2) artifact filtering; and (3) species-level abundance estimation. For data analysis One Codex Application Program Interface (API; v1) embedded within Jupyter notebook (v0.9.6) with python library (v3.8.3) was used. The relative abundance of each microbial species was estimated based on the depth and coverage of sequencing across every available reference genome. Here plots of relative taxonomic abundance, alpha diversity values (Shannon, Chao1, and Simpson), beta diversity with principal co-ordinate analysis (PCoA) on Bray–Curtis distances, and Spearman’s correlation as a heatmap were all generated. Alpha diversity values were subsequently imported into GraphPad Prism (v9.3.1) for graphing.

### Data analysis and statistics

All animal sample size calculations were performed using G*Power3 [47] with a default significance level (α) of 0.05 and power (1-ß) of 0.80. Effect sizes (d and F) were calculated (https://www.psychometrica.de/) based on data obtained from previous unpublished research. Bifidobacterial CFUs were standardised per gram of material and the resulting data was then used for statistical comparison (*t* test) employing GraphPad Prism (v9.3.1). Alpha diversity repeated measures were used to assess statistical significance for each group at each timepoint in comparison to its respective starting timepoint at Day -7, these were completed with GraphPad. Statistical analysis of beta-diversity was performed using a permutational multivariate analysis of variance (PERMANOVA) and analysis of similarities (ANOISM) using Scikit-bio software library [48] within the One Codex application. Results generated from qPCR of host immune and epithelial barrier function were analysed using Kruskal–Wallis test in GraphPad. Histological measurements produced from Fuji (v1.14.0) were also analysed using Kruskal–Wallis and one-way ANOVA within GraphPad.

## Results

### A diet with a high dose of 2’-FL and scGOS/lcFOS improves transient bifidobacterial strain colonization

**Fig. 2:**
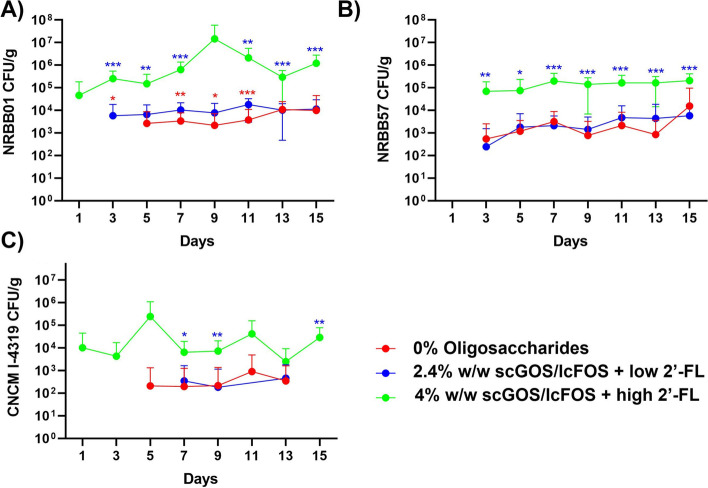
Specific faecal bifidobacterial counts recovered by selective antibiotic plating. Results of 15 days of optimization and clindamycin trial of animals that were supplemented with with/out GOS/FOS prebiotic diet either enriched with low or high dose of 2’-FL and the bifidobacterial mixture. **A** *B. breve* NRBB01, **B** *B. breve* NRBB57, and **C** *B. bifidum* CNCM-I 4319. Values equal to zero are not shown, *n* = 10 (NB. bifidobacterial mix animals not administered with clindamycin are only shown in this graph). Individual values represent the mean (± SD) produced from replicate plating of 10 animals and significance level (multiple *t* test): *, *p* value < 0.05; **, *p* value < 0.01; ***, *p* value < 0.001

The control diet (AIN93G) included either no scGOS/lcFOS/2’-FL or a 2.4% w/w carbohydrate substitution during the optimization trial, this was subsequently increased to 4% w/w (Table S2) during the clindamycin challenge trial. The increase to 4% scGOS/lcFOS/2’-FL was determined after the optimization trial colonization rates for *B. breve* NRBB57 and *B. bifidum* CNCM I-4319 were not significantly increased when compared to the control diet. Figure 2 compares the results of the optimization trial (0% and 2.4% scGOS/lcFOS/2’-FL) and the first 15 days of the clindamycin trial during which animals were fed a 4% scGOS/lcFOS/2’-FL prebiotic diet and treated with the bifidobacterial mix. Faecal plate counts for each individual probiotic strain within the mix treatment revealed that the diet enriched with 4% scGOS/lcFOS/2’-FL resulted in the most significant synergistic impact on the colonization levels for all 3 strains. Of interest, none of the strains were able to remain/persist within the host following the washout period; in fact, without clindamycin treatment all bifidobacterial counts returned to zero (or undetectable) after 48 h from the final gavage. Bifidobacterial plating of the ileum, cecum, and colon tissue/content for each respective cull day (24 h post-final gavage or 7 days post-gavage) yielded CFU/g results for only the autochthonous bifidobacterial community (data not shown).

### Clindamycin murine model—bifidobacterial colonization

**Fig. 3:**
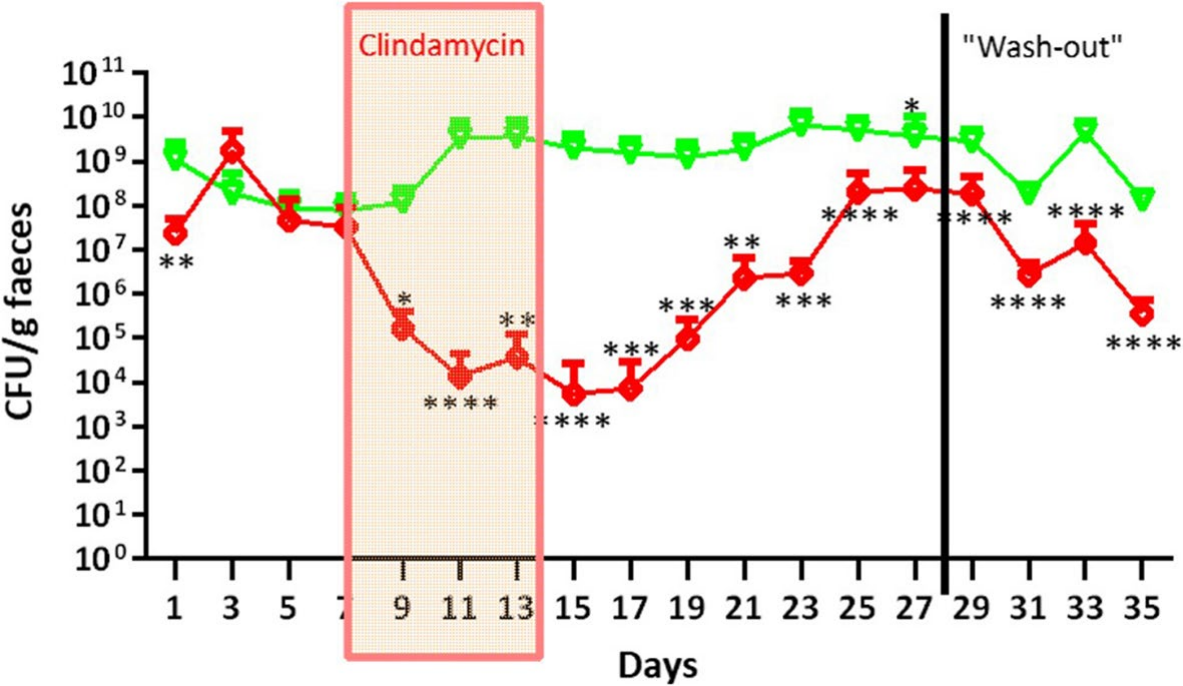
Total *Bifidobacterium* CFU/g faecal plating for mice treated with scGOS/lcFOS/2’-FL diet only. (Red) denotes mice received clindamycin challenge from day 7–14, where (Green) shows mice abstained from antibiotic challenge. Values are plotted on a log10 axis and values equal to zero are not shown, *n* = 10. Individual values represent the mean (± SD) produced from replicate plating of 10 animals and significance level (multiple *t* test): *, *p* value ≤ 0.05; **, *p* value ≤ 0.01; ***, *p* value ≤ 0.001; ****, *p* value ≤ 0.0001

Next, we aimed to assess colonisation of autochthonous *Bifidobacterium* with/out the 7-day antibiotic challenge. Total *Bifidobacterium* counts recovered from plated faecal material for the duration of the Clindamycin Trial were significantly affected by the antibiotic challenge (Fig. 3). There is a clear depreciation of the autochthonous *Bifidobacterium* population, which still had not returned to baseline 3 weeks post-antibiotic treatment. All groups were fed the 4% scGOS/lcFOS/2’-FL diet and it is worth noting that the presence of these bifidogenic carbohydrates was not sufficient to recover the indigenous bifidobacterial community 3 weeks post-antibiotic. No significantly different metabolic end products were observed in the HPLC data generated from ileum and caecum contents (Figures S1 and S2).

### MGS reveals significant recovery of the microbiota upon synbiotic treatment following antibiotic challenge

**Fig. 4:**
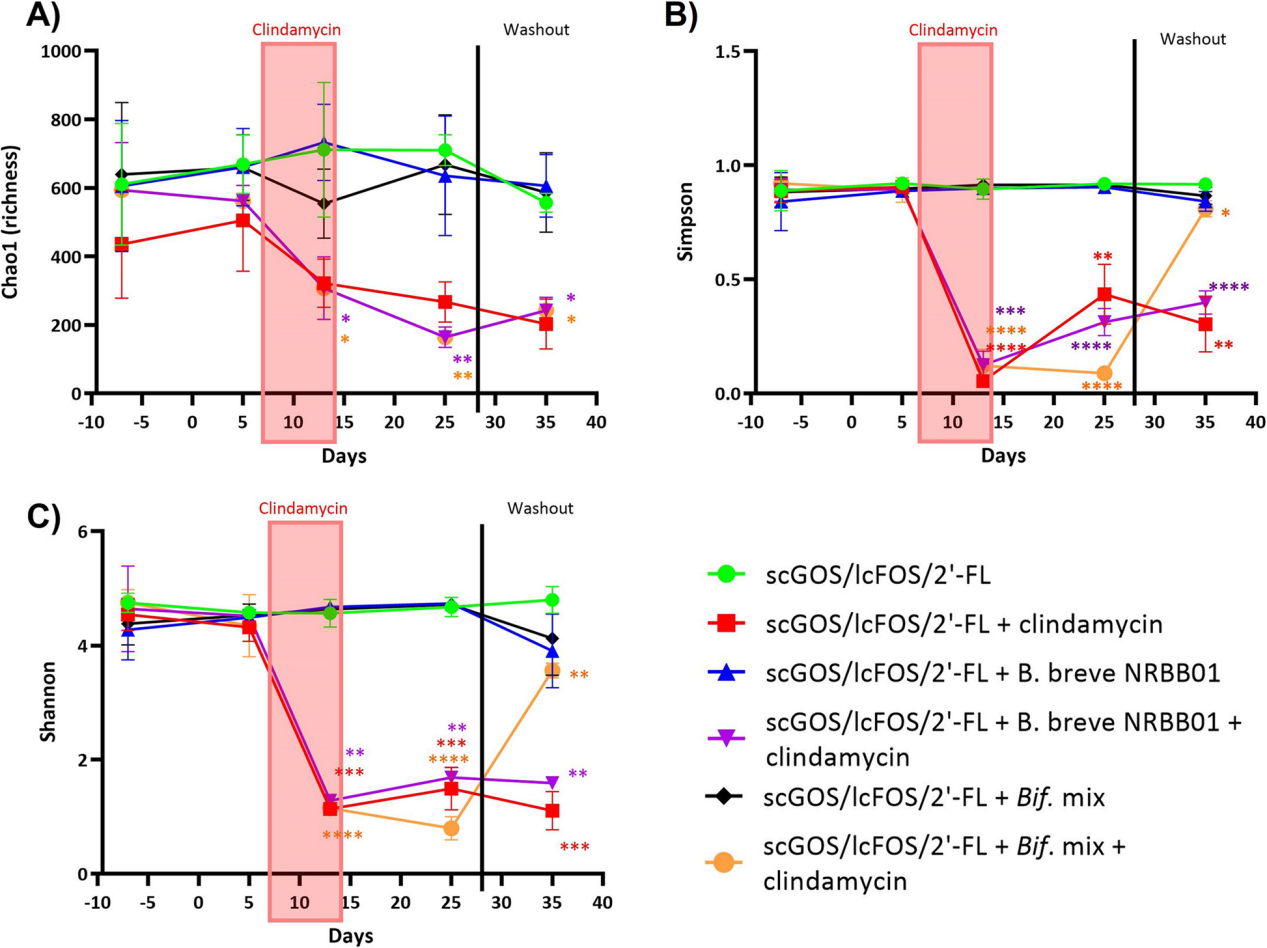
Alpha diversity metrics. **A** Chao1 (aka evenness), **B** Simpson, and **C** Shannon. Individual values represent the mean (± SD) produced from 5 animals/group and statistical comparison are completed between timepoint and its respective starting timepoint at day -7 (significance color corresponds to group). Significance level (multiple *t* test): *, *p* value ≤ 0.05; **, *p* value ≤ 0.01; ***, *p* value ≤ 0.001; ****, *p* value ≤ 0.0001

A subset of samples (*n* = 5/group) from the clindamycin trial were selected to represent the longitudinal shift of the microbiota across the model. Groups were provided the 4% scGOS/lcFOS/2’-FL diet only, or synbiotic mixture of scGOS/lcFOS/2’-FL diet and *B. breve* NRBB01, or bifidobacterial mix were selected for microbiome sequencing. Based on faecal counts of total bifidobacteria (Fig. 3), timepoints selected included those considered to represent “major events” that affected the microbiota. These included the day of arrival from the supplier (pre-diet; day -7), 14 days following prebiotic diet (day 5), end of antibiotic challenge (day 13), end of gavage period and 14 days post-antibiotics (day 25), and finally end of the “washout” period (day 35). MGS from mouse faecal pellets gDNA, from 30 mice across 5 timepoints, resulted in a total of 1.3 billion reads passing filtering with a median of 14,027,447 ± 3,091,316 reads/sample. Shannon, Chao1, and Simpson Index, markers of microbiota alpha diversity, demonstrated a steep decrease in diversity upon antibiotic treatment. Interestingly, alpha diversity was only restored (returned to baseline values; Simpson and Shannon) 21 days post-antibiotic exposure for animals that received scGOS/lcFOS/2’-FL + bifidobacterial mix, this was not observed in groups that were fed with only the prebiotic or synbiotic formulation with single strain *B. breve* NRBB01 (Fig. 4).

**Fig. 5:**
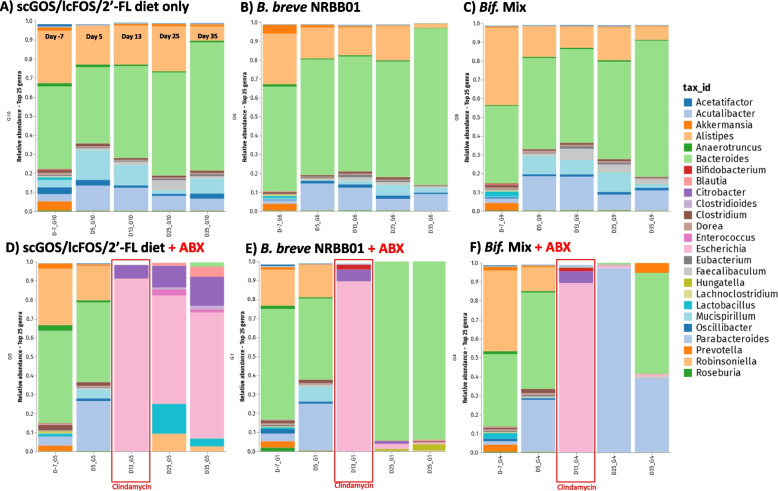
Relative abundance of the top 25 genera at day -7, 5, 13, 25, and 35 across the 3 intervention groups supplemented with **A** scGOS/lcFOS/2’-FL diet only **B** scGOS/lcFOS/2’-FL and B. breve NRBB01 and, **C** scGOS/lcFOS/2’-FL and the *Bifidobacterium* strain Mix without clindamycin treatment. Groups treated with clindamycin between day 7 and 14 (marked by red box) include groups supplemented with **D** scGOS/lcFOS/2’-FL diet only, **E** scGOS/lcFOS/2’-FL and B. breve NRBB01, and **F** scGOS/lcFOS/2’-FL and the *Bifidobacterium* strain mix. *N* = 5/group; unassigned genera have been excluded, Figure S3 details the taxonomic composition for individual animals with unassigned genera included

In animals not treated with clindamycin, taxonomic composition profiles (Fig. 5A; Figure S3 and S4) revealed that supplementation of scGOS/lcFOS/2’-FL to the diet most notably affects the relative abundance of *Parabacteroides* and *Mucispirillum* and groups fed with synbiotics generally share and maintain a consistent profile predominately of *Alistipes*, *Bacteroides* and *Parabacteroides* (Fig. 5B, C). These data, along with non-significant alterations in alpha-diversity from day -7, suggest that addition of bifidobacteria to the scGOS/lcFOS/2’-FL diet did not result in a significant impact on the host microbiota composition in the absence of antibiotic challenge.

**Fig. 6:**
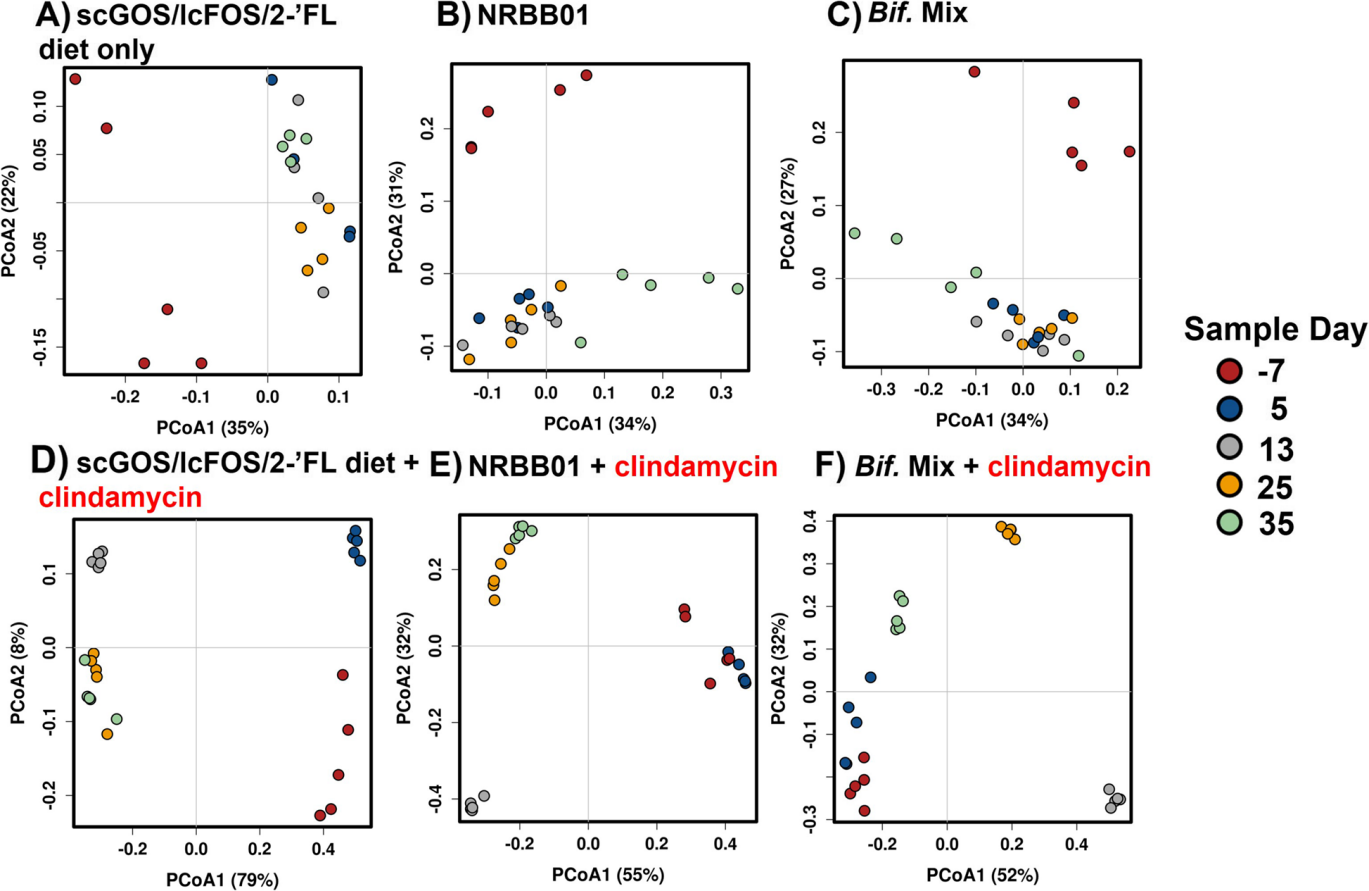
PCoA plots of beta diversity for non-antibiotic treated groups **A** scGOS/lcFOS/2’-FL diet only, **B** scGOS/lcFOS/2’-FL +* B. breve* NRBB01, and **C** scGOS/lcFOS/2’-FL + *Bifidobacterium* Mix. Antibiotic challenged groups include **D** scGOS/lcFOS/2’-FL only, **E** scGOS/lcFOS/2’-FL + *B. breve* NRBB01, and **F** GOS/FOS/2’-FL + *Bifidobacterium* Mix. Data points are colour coded by faecal collection day -7 (animal arrival), day 5 (1 week on scGOS/lcFOS/2’-FL diet), 13 (end of clindamycin challenge), 25 (2 weeks post-clindamycin), and 35 (3 weeks post clindamycin)

In all groups challenged with clindamycin the microbiota exhibited a dramatic shift towards a community dominated by *Citrobacter* and *Escherichia* observed at day 13 (Figure S4C and S4F, respectively). Twenty-one days following treatment completion the composition of the scGOS/lcFOS/2’-FL diet group only still retained a similar microbial profile (Fig. 5D), accompanied by an increase of *Lactobacillus*, *Enterobacter, Enterococcus* and *Clostridioides* (Figure S4). These results suggest the supplementation with scGOS/lcFOS/2’-FL alone was not enough to restore a normal/balanced microbiota. In contrast, animals treated with clindamycin and supplemented with synbiotic *B. breve* NRBB01 and scGOS/lcFOS/2’-FL became re-colonised and dominated by *Bacteroides*, after treatment. This was maintained even after the “washout” period (Fig. 5E and Figure S4B). Within this same group, upon cessation of *B. breve* NRBB01 gavage, *Hungatella hathewayi* appears to fill the ecological niche. However, the most striking result was the synbiotic bifidobacterial mix treatment (Fig. 5F and Figure S3F), by day 25 animals were predominately re-colonized by *Parabacteroides* (Figure S4H), and after the “washout” period (day 35) the microbiota appeared more diverse with a recovery of genera present before challenge (day 5), namely *Parabacteroides*, *Bacteroides*, and *Akkermansia* being the most prominent. Beta diversity for each group over the time course confirmed this observation with animals provided synbiotic bifidobacterial mixture 21 days post-antibiotic challenge (day 35) cluster closer with those of baseline animals (day 5, Fig. 6F; ANOISM *p* = 0.001 and PERMANOVA *p* = 0.001) than any other clindamycin group. The PCoA also shows that the non-antibiotic groups cluster closely together once animals commence the AIN93G-scGOS/lcFOS/2’-FL diet, compared to day − 7 (day animals are received; Fig. 6A, C). Of the challenge group, the prebiotic only group (Fig. 6D; ANOISM *p* = 0.001 and PERMANOVA *p* = 0.001) demonstrated the effect clindamycin has on microbial diversity with animals forming distinct clusters away from baseline (day − 7 and 5) following challenge (day 13) and the shift away from baseline is maintained for the duration of the trial (day 25 and 35). In comparison, clindamycin-administered mice treated with *B. breve* NRBB01 synbiotic (Fig. 6E; ANOISM *p* = 0.001 and PERMANOVA *p* = 0.001) harbour distinct profiles from day 25 and 35, post-antibiotic, which clusters away from baseline. Individual statistical comparisons (PERMANOVA) between each timepoint for each respective group are detailed in Table S4. Collectively, our results demonstrate that the synbiotic effect of the bifidobacterial mix + scGOS/lcFOS/2’-FL supplemented diet was the most effective treatment for initiating a recovery of the microbiota. While beta diversity clustering suggests that 21 days post-antibiotic challenge (day 35) microbiota begins to resemble the profile before clindamycin perturbation (day 5) the difference is still significant (PERMANOVA *p* = 0.011). Future work will extend the monitoring period to determine if and when this synbiotic mix is capable of total host gut microbiota restoration in comparison to no synbiotic intervention.

### Antibiotic effects on the host and mitigation of these effects by a synbiotic

**Fig. 7:**
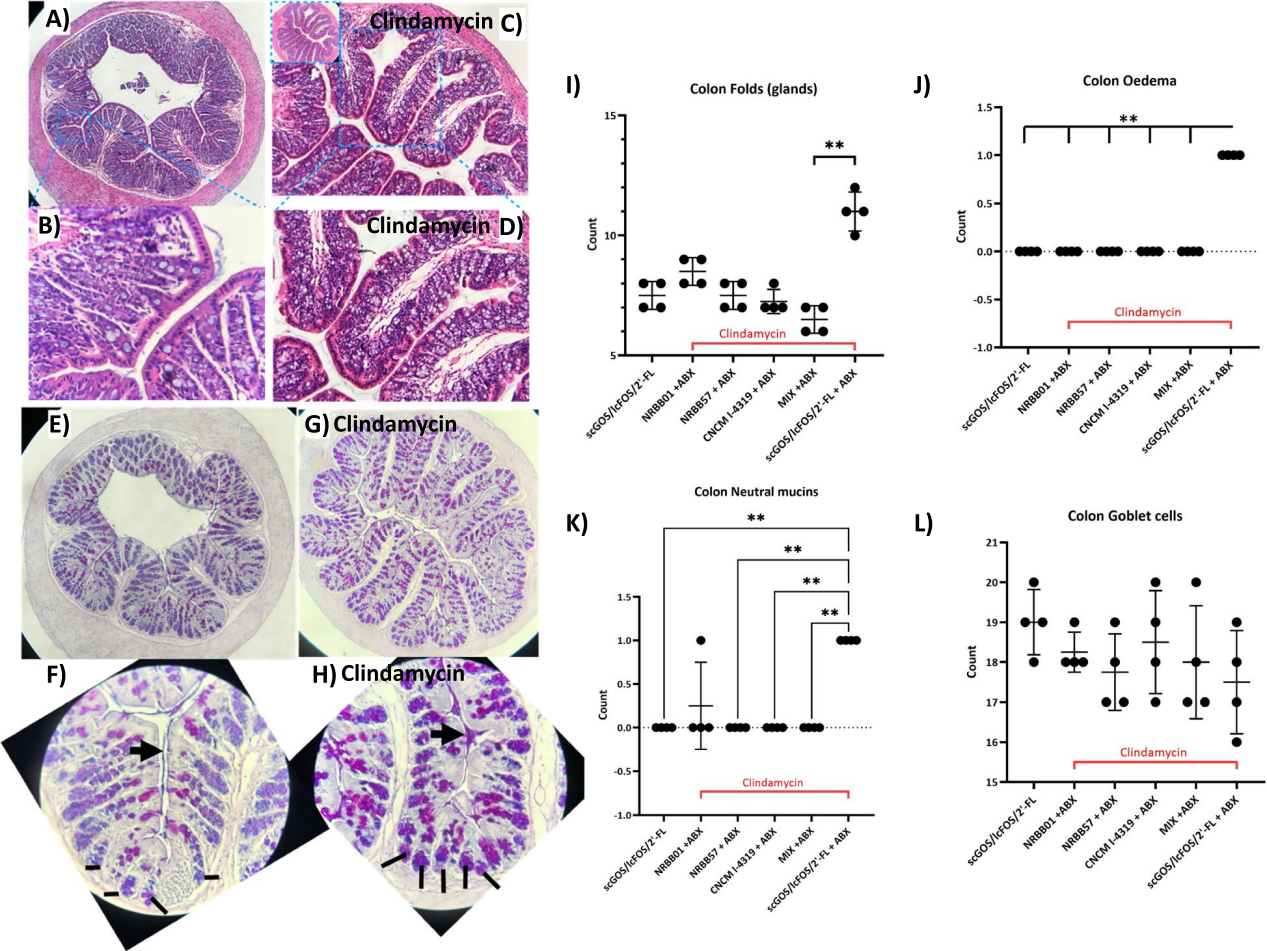
Clindamycin induced intestinal changes remain in mice 14 days after cessation (day 29). **A**–**D** Changes in histopathological appearance by H&E staining are evident in prebiotic diet only groups without (**A**, **B**) and with clindamycin treatment (**C**, **D**) with increased colonic glands and oedema at crypt and colonic fold base. **E–H** Alcian Blue/PAS staining of neutral (pink-red), acidic (blue), mixed (purple) mucins reveal increased staining of neutral mucins following antibiotic treatment (**G**, **H**) throughout the colon. This is evident from the crypt to fold tip (apex), see arrows highlighting mucus staining and lines illustrating the colonic crypt (**F**, **H**). Histological scoring of the **I** colonic folds number (glands), **J** oedema, **K** neutral mucin staining, **L** and goblets cells were measured with at least 5 per mouse compared to the prebiotic diet only group. Data are expressed as means ± SD. *n* = 4 per group. Mean values were significantly different between the groups: ****P < 0.0001

Clindamycin administration resulted in increased cecum length 2 weeks post-antibiotic challenge (day 29), compared to non-antibiotic groups (Figure S5). The 35-Day cohort cecum dimensions were reduced in comparison to 29-day, but still had not returned to “baseline” (Supplementary Figure S5). Increased cecum length is likely due to water retention [49], observed oedema caused by immune influx (see below [50]), and aggravation of the compromised microbiota as evidenced by our MGS. Microscopic evaluation of intestinal tissue was performed to assess epithelial integrity (Fig. 7A–D) and acidic and neutral mucin distribution (Fig. 7E–H). Ileum histology show mild oedema and villi/colonic crypt irregularities following clindamycin treatment, which was less pronounced for animals provided synbiotic formulation scGOS/lcFOS/2’-FL + *B. breve* NRBB01 or the scGOS/lcFOS/2’-FL + bifidobacterial mix. In the colon, at day 29, clindamycin increased colonic folds, oedema and distribution of neutral mucin staining in the scGOS/scFOS/2’-FL group.

While there was no evident change in goblet cell numbers or mucus thickness, neutral mucin distribution changed from the apical tip of the colonic folds in the non-clindamycin group fed 4% scGOS/scFOS/2’-FL to being distributed throughout the colonic fold from the crypt base to apical tip (Fig. 7F–H). This effect was abrogated by synbiotic scGOS/lcFOS/2’-FL + bifidobacterial mix supplemented groups after clindamycin therapy. No discernible difference between the scGOS/lcFOS/2’-FL only and clindamycin treated group was evident at day 35 by microscopy (Figure S6).

Pro-inflammatory (TNF-α, IL-1β, IFN-g, IL-6), anti-inflammatory (IL-10) cytokines (Figure S7–S8) and gut integrity (Muc2, CLDN-1, CLDN-2, DEFA5) host markers (Figure S9–S10) were assessed from colon tissue collected from antibiotic trial endpoints (day 29 endpoint was collected after the last gavage or day 35 endpoint which represented a 7-day *Bifidobacterium* treatment washout). Animals which had not received antibiotic challenge did not exhibit significant changes in IL-1β, IL-6, IL-10, TNF-α, and IFN-g expression between prebiotic only group and bifidobacterial synbiotic groups. Clindamycin increased IL-1β at 29-day endpoint for animals given only the scGOS/lcFOS/2’-FL diet, while both scGOS/lcFOS/2’-FL + *B. breve* NRBB01 and scGOS/lcFOS/2’-FL + *B. bifidum* CNCM-I 4319 reduced IL-1β expression (Figure S7A). IL-6 was decreased after synbiotic feeding in clindamycin with GOS/FOS/2’-FL + *B. breve* NRBB01 (day 29) and *B. breve* NRBB57 (day 35) having the most pronounced effect (Figure S7B and E). TNF-α appears to also be increased by clindamycin treatment at day 35, possibly because of increased abundance of “pathogenic” species, and synbiotic treatment with *B. bifidum* CNCM I-4319 was most notable at suppressing production of this cytokine at the 35-day endpoint (Figure S8A and C). Our results also suggest IL-10 is induced by the administered synbiotic with bifidobacterial strain mix at day 29 (Figure S7C). However, in synbiotic treatment groups at day 35 there was a significant decrease for *B. bifidum* CNCM-I 4319 (Figure S7F). In comparison to clindamycin, animals that were fed only the scGOS/lc/FOS/2’-FL diet had decreased IFN-g versus animals not given antibiotic or bifidobacterial gavage (Figure S8B and D). When comparing IFN-g in groups provided synbiotic treatment there was a marginal increase in animals administered *B. bifidum* CNCM-I 4319 or the *Bifidobacterium* mix at day 29, and at day 35 *B. breve* NRBB01 and *B. breve* NRBB57 were also increased compared to antibiotic scGOS/lcFOS/2’-FL group (Figure S8B and D). Muc2 relative expression was decreased at day 29 for synbiotic bifidobacterial mix clindamycin-treated animals (Figure S9A). DEFA-5 and CLDN-2 were unremarkable for all animals and timepoints (Figure S9 and S10). However, transmembrane tight junction protein claudin-1 (Cldn-1) was increased in the scGOS/lcFOS/2’-FL + clindamycin group at 29-day endpoint (Figure S10). This increase was significant to not only the non-clindamycin group (*B. breve* NRBB01), but also the synbiotic treatments *B. breve* NRBB57, *B. bifidum* CNCM I-4319 and the bifidobacterial mix at day 29 (Figure S10A), these values returned to “baseline” at day 35 (Figure S10C).

### Genera level correlations to host factors

**Fig. 8:**
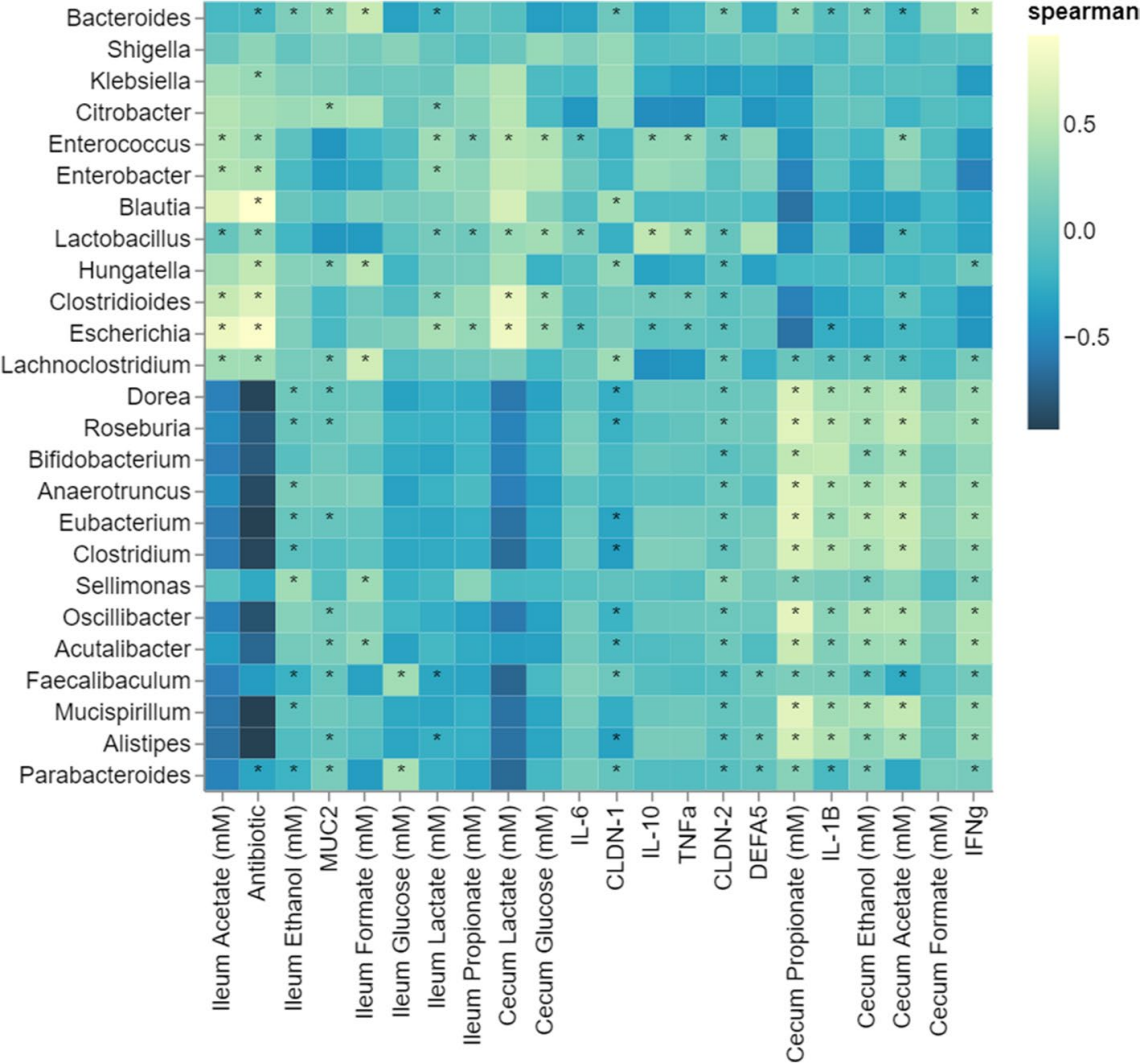
Associations (Spearman correlation) between the top 25 genera identified of all samples and host factors (histological scores, barrier function, immune marker expression, and short-chain fatty acids from the cecum and ileum)

Finally, we conducted Spearman correlations of the MGS data and the metabolomic, histological and qPCR results of host immune and epithelial markers for the day 35 endpoint. The host and metabolic data for matched animals with reported MGS were only included in this analysis (Fig. 8). Overall, this analysis depicts 2 distinct profiles which reflect the taxonomic composition in animals with and without antibiotic challenge. Genera negatively associated with clindamycin were associated with metabolite production (propionate, ethanol, acetate, and formate) in the cecum. Conversely, genera positively associated with clindamycin are correlated with ileum metabolites. This analysis also demonstrates that there is a positive relationship between microbial genera *Escherichia*, *Clostridioides*, *Enterococcus*, and *Lactobacillus* and TNF-α, these genera were also associated with the clindamycin treatment and there is a striking enrichment of these genera upon antibiotic challenge (Fig. 5D–F).

## Discussion

The restorative properties of synbiotics for compromised GI microbiota because of premature birth/delivery method has been previously demonstrated [29, 51]; however, the ability of different synbiotics to restore an antibiotic-challenged microbiota remains unclear. This study aimed to assess a synbiotic mixture of prebiotics and infant type bifidobacteria for its ability to rescue a compromised or perturbed microbiota in early life (i.e., C-section, preterm, or antibiotics). We report that murine GIT microbiota resilience can be supported by synbiotic supplementation of a prebiotic mix, (scGOS/lcFOS) enriched with HMO (2’-FL), in combination with one or more infant-type bifidobacterial strains upon challenge with clindamycin.

Previous studies in animals and humans attempting to recover antibiotic perturbed microbiotas with probiotic formulations [26, 27] concluded that microbial re-establishment could likely be delayed as a result of the probiotic administration [26]. However, most patients are unlikely to receive multiple antibiotics as “standard” treatment. In addition to the substantial microbiota remodelling that occurs as a result of antibiotics, there is increased risk of secondary GIT bacterial infection, one example being *C. difficile*-associated diarrhoea [52, 53]. *C. difficile* is more common in formula-fed infants [54–56], while *C. difficile*-associated diarrhoea may be considered rare, although increasing, it is often associated with antibiotic use [57]. The clindamycin animal model we employed is representative of an infantile compromised microbiota as a result of environmental challenges such as antibiotic use, mode of delivery, and birth prematurity. Previous work demonstrated that clindamycin negatively affects the Bacteroidetes phylum, whereas the Firmicutes phylum remains largely unchanged in relative abundance, while Proteobacteria expands dramatically [58]. We observed a similar result with the remodelled microbiota profile appearing adverse or even potentially pathogenic, dominated by *Escherichia coli*, *Clostridioides difficile*, *Citrobacter* spp., and *Shigella* spp.

Following birth, the GIT microbiota from vaginally born infants typically consists of *Bacteroides*, *Bifidobacterium*, *Parabacteroides*, and some *Escherichia/Shigella* species [2] and disruptions of this community early in life has been shown to have long lasting negative health effects, such as asthma, obesity, metabolic diseases, neurocognitive development, neonatal morbidity, and mortality [5, 15, 59]. While previous studies in both animal models and new-borns have reported on the protective benefits of commensal strains against pathogenic challenge [14, 28, 58, 60–62], few have also commented on the recovery of the microbiota [31, 63–65]. To address this, we first worked to improve colonization of our bifidobacterial strains by dietary supplementation [38] with scGOS/lcFOS/2’-FL. Previous research demonstrated positive effects of supplementing infant formula with GOS and FOS, including reduced occurrence of gastroenteritis and potential reduction of respiratory infections [66, 67], modulation of the immune response [68–70], and suppression of *C. difficile* [71]. 2’-FL is the dominant HMO present in most lactating mothers and benefits the host through reducing necrotising enterocolitis in preterm birth (NEC) by increasing endothelial nitric oxide synthase to enhance intestinal blood flow [72–75]. Particular members of *Bifidobacterium* genus dominate breast-fed babies (such as *B. longum* subsp. *infantis* and *B. bifidum*) and encode enzymatic machinery necessary to utilize HMOs [76, 77], and as such HMOs are used as a selective and supportive carbohydrate [78]. Indeed, we observed that supplementing the control diet with 4% scGOS/lcFOS/2’-FL improved the colonization efficiency of each of strains. Addition of scGOS/lcFOS/2’-FL alone was not sufficient to restore the GI microbiota following antibiotic challenge. Synbiotic treatment of scGOS/lcFOS/2’-FL and *B. breve* NRBB01 resulted in a partially recovered profile, shifting from Proteobacteria dominated to a recovery and domination of *Bacteroides* from antibiotic completion until the end of the trial. Although alpha diversity did not return to baseline, there was a marked suppression of *E. coli*, *C. difficile*, *Citrobacter*, and *Shigella* spp. Similar promotion of Bacteroidetes was reported by Crouzet L, Derrien M, Cherbuy C, Plancade S, Foulon M, Chalin B, van Hylckama Vlieg JET, Grompone G, Rigottier-Gois L and Serror P [79] with *Lactobacillus paracasei* supplementation. Promisingly, we found that the synbiotic with bifidobacterial mix, while initially dominated by *Proteobacteria* after the antibiotic period, subsequently shifted to a more diverse profile. While we observed a partial recovery of the microbiota this may be a result of testing human-adapted *Bifidobacterium* in animal model and/or the premature termination of the trial before a full recovery could be observed. However, we can conclude that treatment with a synbiotic consisting of scGOS/lcFOS/2’-FL and single strain *B. breve* NRBB01 is only partly sufficient to promote recovery of the microbiota, while the effect of the synbiotic mixture of scGOS/lcFOS/2’-FL, *B. breve* NRBB01, *B. breve* NRBB57, and *B. bifidum* CNCM I-4319 was stronger.

Regarding the host response to clindamycin, long-term disruption to intestinal function was still evident 14 days post-antibiotic, with colonic oedema still evident and increased neutral mucin secretion. Effects of antibiotics on mucin and mucus secretion has been previously observed, suggesting increased neutral mucins allow pathogenic gram-negative bacteria to thrive [43, 44]. The restoration to histological normal appearance by synbiotic supplementation supports the trend towards restoration of the GIT microbiota. RT-qPCR analysis of the synbiotic treatment groups during clindamycin indicated increased IL-10 and IFN-g, suggestive of T cell recruitment, and possibly even T regulatory cells induction, possibly in response to the “pathogenic” microbiota shift observed following treatment. Host factor DEFA-5 is abundant in neutrophil granules and is also found in mucosal epithelium, including the intestine, we observed no significant impact on this from either clindamycin or synbiotic treatment. It should also be noted that our dosage of bifidobacterial strains was relatively high (1 × 10^9^ CFU) for the animal model and translation to human infants; however, it did allow us to confirm that there were no adverse host physiological responses to this synbiotic combination. Overall, the animal model of clindamycin facilitates high throughput cost effective assessment of synbiotics modulating capacity of the microbiota and induction of host epithelial changes. Future studies using this model could include a sample point closer to antibiotic challenge and conduct immunohistochemistry to assess recruitment and infiltration of immune cells. Furthermore, it will be very interesting to investigate specific individual variations within treatment groups to examine the effects of coprophagic activity within co-housed mice and microbiota recovery. Next steps for our research will see the validation of the synbiotic efficacy within human infants at times of microbiota vulnerability (i.e., antibiotic challenge, premature birth, caesarean birth).

## Concluding remarks

Previous work employing single [27] and multi-strain [26] probiotics have demonstrated that formulations and type/combination of antibiotics dictates the success of baseline microbiota re-establishment and conferral of host benefits. We aimed to investigate potential effects of prebiotics alone and synbiotics on a disturbed microbiota to restore the GI microbiota. Our study supports a rapid and profound effect on partial microbial restoration with a bifidobacterial mixed synbiotic. Future work should aim to examine whether this synbiotic formulation can restore the microbiota under alternative circumstances (e.g., different antibiotics, pre-term birth).

## Supplementary Information

**Additional file 1:** **Table S1.** Strains used in this study. **Table S2.** Diet formulation manufactured by SSNIFF (www.ssniff.com). **Table S3.** Primers used in this study. **Table S4.** PERMANOVA statistics between microbiota sequence timepoints of beta diversity matrices for each treatment group. N=5/group. **Figure S1.** Ileum content HPLC VFA results for A) groups culled on Day 29, and B) Replicate groups culled on Day 35. **Figure S2.** Cecum content HPLC VFA results for A) groups culled on Day 29, and B) replicate groups culled on Day 35. **Figure S3.** Relative abundance of the top 25 genera at Day -7, 5, 13, 25 and 35 across the 3 intervention groups supplemented with A)scGOS/lcFOS/2’-FL and *B.breve* NRBB01 B) scGOS/lcFOS/2’-FL and the *Bifidobacterium* strain Mix and C) scGOS/lcFOS/2’-FL diet only, without clindamycin treatment. Groups treated with clindamycin between Day 7-14 include groups supplemented with D) scGOS/lcFOS/2’-FL and B. breve NRBB01 E) scGOS/lcFOS/2’-FL and the *Bifidobacterium* strain Mix and F) scGOS/lcFOS/2’-FL diet only. N=5/group. **Figure S4.** Line plot of the relative abundance of key genera A) *Akkermansia*, B) *Bacteroides*, C) *Citrobacter*, D) *Clostridioides*, E) *Enterococcus*, F) *Escherichia*, G) *Lactobacillus*, H) *Parabacteroides* at Days -7, 5, 13, 25 and 35. Each line represents a treatment group with and without antibiotic treatment (clindamycin) between Day 7-14 (*B. breve *NRBB01, *Bifidobacterium* strain Mix and scGOS/lcFOS/2’-FL diet only), without clindamycin treatment. Clindamycin treatment occurred between Day 7-14; n=5/group. **Figure S5.** Cecum dimensions for replicate groups A (29 Day) and B (35 Day) demonstrate a difference between groups treated with and without antibiotics. Cecums of animals receiving clindamycin treatment are larger in length, which is more noticeable for Group A (cull Day 29) which was culled 2 weeks post antibiotic treatment (*n* = 5). Group B (cull Day 35) had an n=1 and PBS+ABX control was not imaged. Therefore, these results are only observational and not appropriate for statistical analysis. **Figure S6.** Histological scoring found no significant differences, at Day 35 (3 weeks post antibiotic), of the colonic folds number (glands), edema, neutral mucin staining, and goblets cells (I-L) were measured with at least 5 per mouse. Data are expressed as means ± SD. n= 4 per group. Mean values were significantly different between the groups: *P<0.05, **P<0.01, *** *P*<0.001. **Figure S7.** Immune markers A) IL-1b, B) lL-6, and C) IL-10 qPCR relative expressions at Day 29 and D) IL-1b, E) lL-6, and F) IL-10 at Day 35. Data are expressed as Log 2 transformed ± SD; n= 5 per group. Mean values were significantly different between the groups: *P<0.05, **P<0.01, *** *P*<0.001. **Figure S8.** Immune markers A) TNF-α and B) IFN-g qPCR relative expressions at Day 29 and C) TNF-α D) IFN-g at Day 35. Data are Log 2 transformed ± SD; n= 5 per group. Mean values were significantly different between the groups: *P<0.05, **P<0.01, *** *P*<0.001. **Figure S9.** Epithelial barrier markers A) Muc2, and B) DEFA5 at Day 29 qPCR relative expressions and C) Muc2, D) DEFA5 at Day 35. Data is Log 2 transformed ± SD; n= 5 per group. Mean values were significantly different between the groups: *P<0.05, **P<0.01, *** *P*<0.001. **Figure S10.** Epithelial barrier markers A) CLDN-1 and A) CLDN-2 qPCR relative expressions at Day 29 and C) CLDN-1, D) CLDN-2 at Day 35. Log 2 transformed data as ± SD; n= 5 per group. Mean values were significantly different between the groups: *P<0.05, **P<0.01, *** *P*<0.001.

## Data Availability

Raw sequence data is available and has been deposited at NCBI SRA under the BioProject accession number PRJNA732472.
