# Supplementary material for: A synbiotic mixture of selected oligosaccharides and bifidobacteria assists murine gut microbiota restoration following antibiotic challenge
Source: Microbiome. 2023 Aug 2;11:168. doi: 10.1186/s40168-023-01595-x (PMC10394833; doi:10.1186/s40168-023-01595-x)
Supplement: Supplementary file 2 — Additional file 1: Table S1. Strains used in this study. Table S2. Diet formulation manufactured by SSNIFF (www.ssniff.com). Table S3. Primers used in this study. Table S4. PERMANOVA statistics between microbiota sequence timepoints of beta diversity matrices for each treatment group. N=5/group. Figure S1. Ileum content HPLC VFA results for A) groups culled on Day 29, and B) Replicate groups culled on Day 35. Figure S2. Cecum content HPLC VFA results for A) groups culled on Day 29, and B) replicate groups culled on Day 35. Figure S3. Relative abundance of the top 25 genera at Day -7, 5, 13, 25 and 35 across the 3 intervention groups supplemented with A)scGOS/lcFOS/2’-FL and B.breve NRBB01 B) scGOS/lcFOS/2’-FL and the Bifidobacterium strain Mix and C) scGOS/lcFOS/2’-FL diet only, without clindamycin treatment. Groups treated with clindamycin between Day 7-14 include groups supplemented with D) scGOS/lcFOS/2’-FL and B. breve NRBB01 E) scGOS/lcFOS/2’-FL and the Bifidobacterium strain Mix and F) scGOS/lcFOS/2’-FL diet only. N=5/group. Figure S4. Line plot of the relative abundance of key genera A) Akkermansia, B) Bacteroides, C) Citrobacter, D) Clostridioides, E) Enterococcus, F) Escherichia, G) Lactobacillus, H) Parabacteroides at Days -7, 5, 13, 25 and 35. Each line represents a treatment group with and without antibiotic treatment (clindamycin) between Day 7-14 (B. breve NRBB01, Bifidobacterium strain Mix and scGOS/lcFOS/2’-FL diet only), without clindamycin treatment. Clindamycin treatment occurred between Day 7-14; n=5/group. Figure S5. Cecum dimensions for replicate groups A (29 Day) and B (35 Day) demonstrate a difference between groups treated with and without antibiotics. Cecums of animals receiving clindamycin treatment are larger in length, which is more noticeable for Group A (cull Day 29) which was culled 2 weeks post antibiotic treatment (n = 5). Group B (cull Day 35) had an n=1 and PBS+ABX control was not imaged. Therefore, these results are only observat [file 40168_2023_1595_MOESM1_ESM.docx]

**Supplementary material – murine HMO/ABX manuscript**

**Table S1.** Strains used in this study.

| **Strain** | **Associated plasmid** | **Selective pressure** |
| --- | --- | --- |
| *Bifidobacterium breve* NRBB01 | pSKEM (35) | Erythromycin 50 µg/mL |
| *Bifidobacterium breve* NRBB57 | pPKCM (35) | Chloramphenicol 25 µg/mL |
| *Bifidobacterium bifidum* CNCM I-4319 | pDM1 (34) | Spectinomycin 400 µg/mL |

**Table S2.** Diet formulation manufactured by SSNIFF ([www.ssniff.com](http://www.ssniff.com)).

|  |  | **AIN93-G** | **AIN93-G** | **AIN93-G** |
| --- | --- | --- | --- | --- |
| **g/ 1 kg feed** |  | **Control** | **2.4% scGOS/lcFOS/HMOS (9:1:2)** | **4% scGOS/lcFOS/HMOS (9:2:9)** |
| Functional |  | **g/kg feed** | **g/kg feed** | **g/kg feed** |
| **Carbs** | **Raw Material names** |  |  |  |
| Cornstarch | C gel 03401 | 397.49 | 376.43 | 366.96 |
| *Moisture* |  | *47.70* | *45.17* | *44.04* |
| *Carbohydrates* |  | *349.39* | *330.88* | *322.56* |
| Dextrinized cornstarch | Maltodextrin MD1925 WS | 132.0 | 125.0 | 121.9 |
| *Moisture* |  | *6.6* | *6.3* | *6.1* |
| *Carbohydrates* |  | *125.4* | *118.8* | *115.8* |
| Sucrose | Sugar Melis | 100.0 | 94.7 | 92.3 |
| *Moisture* |  | *0.1* | *0.1* | *0.1* |
| *Carbohydrates* |  | *99.9* | *94.6* | *92.2* |
| **Fibre** |  |  |  |  |
| Fibre source (cellulose) | Arbocel B800 | 50.00 | 47.35 | 46.16 |
| *Moisture* |  | *0.25* | *0.24* | *0.23* |
| *Fibre* |  | *49.75* | *47.11* | *45.93* |
| GOS Vivinal syrup (45% GOS) | Galacto oligosaccharide syrup | 0.00 | 40.00 | 40.00 |
| *Moisture* |  | *0.00* | *10.00* | *10.00* |
| *Fibre* |  |  | *18.00* | *18.00* |
| *Lactose* |  | *0.00* | *6.00* | *6.00* |
| *Other carbohydrates* |  | *0.00* | *6.00* | *6.00* |
| Inulin HP (lcFOS) (97% FOS fibre) | Inulin Fibre | 0.00 | 2.06 | 4.12 |
| *Moisture* |  | *0.00* | *0.06* | *0.12* |
| *Fibre* |  |  | *2.00* | *4.00* |
| 2-FucosylLactose (93.4% OS) |  | 0.00 | 4.16 | 19.27 |
| *Fibre* |  |  | *4.16* | *18.00* |
| *Lactose* |  | *0.00* | *0.04* | *0.00* |
| *Moisture* |  | *0.00* | *0.08* | *1.21* |
| **Protein** |  |  |  |  |
| Casein | Acid casein | 200.0 | 200.0 | 200.0 |
| *Moisture* |  | *19.0* | *19.0* | *19.0* |
| *Fat* |  | *1.6* | *1.6* | *1.6* |
| *Protein* |  | *175.6* | *175.6* | *175.6* |
| *Lactose* |  | *0.2* | *0.2* | *0.2* |
| L-cystine | L-Cystine | 3.0 | 3.0 | 3.0 |
| **Fat** |  |  |  |  |
| Soybean oil | Soy oil non-GMO | 70.0 | 70.0 | 70.0 |
| *Fat* |  | *70.0* | *70.0* | *70.0* |
| **Others** |  |  |  |  |
| Mineral mix | Mineral mix AIN 93G | 35.0 | 35.0 | 35.0 |
| *Moisture* |  | *0.4* | *0.4* | *0.4* |
| *Carbohydrates* |  | *7.7* | *7.7* | *7.7* |
| Vitamin mix | Vitamin mix AIN 93VX | 10.0 | 10.0 | 10.0 |
| *Moisture* |  | *0.0* | *0.0* | *0.0* |
| *Carbohydrates* |  | *9.8* | *9.8* | *9.8* |
| Choline bitartrate | Choline bitartrate | 2.50 | 2.50 | 2.50 |
| *Moisture* |  | *0.01* | *0.01* | *0.01* |
| TBHQ | Tert-butylhydroquinone | 0.014 | 0.014 | 0.014 |
| **Nutritional Value** |  |  |  |  |
|  | Total weight | 1000.0 | 1010.2 | 1011.2 |
|  | Dry mass | 922.0 | 925.1 | 926.0 |
|  | Protein | 175.6 | 175.6 | 175.6 |
|  | Total Carbohydrates | 642.1 | 645.2 | 646.2 |
|  | Lactose | 0.2 | 6.2 | 6.2 |
|  | Fat | 71.6 | 71.6 | 71.6 |
|  | Dietary Fibre insoluble | 49.8 | 47.1 | 45.9 |
|  | Dietary Fibre soluble | 0.0 | 24.2 | 40.0 |
|  | Kcal | 3810.7 | 3805.3 | 3809.0 |

**Table S3.** Primers used in this study.

| Target gene | Gene target |  | Primer sequence (5’-3’) | Reference |
| --- | --- | --- | --- | --- |
| NRBB01 | NRBB01_0563 | F | GTGCGCCATGTTGCTCTCG | This study |
| NRBB01 |  | R | GTGCCGTTGTCCATGTCGTC |  |
| NRBB57 | NRBB57_1070 | F | CCGACGGCTCATGAACAGGA | This study |
| NRBB57 |  | R | CCATCGAACGCTGCGGAATC |  |
| CNCM | CNCMI4319_0149 | F | ATTGTCGTTCCCGTTCCTCC | This study |
| CNCM |  | R | TACTCGTACTGCTTGCGACG |  |
| BifITS | 23S | F | CGTGTGAAAGTCCATCGCT | (1) |
| BifITS |  | R | GTCTGCCAAGGCATCCACCA |  |
| β-actin | Host housekeeper protein- qPCR control | F | CTAAGGCCAACCGTGAAAAG | (2) |
|  |  | R | ACCAGAGGCATACAGGGACA |  |
| IL-1β | Interleukin 1 beta  Pro-inflammatory cytokine | F | AGTTGACGGACCCCAAAAG | (2) |
|  |  | R | AGCTGGATGCTCTCATCAGG |  |
| IL6 | Interleukin 6  Pro-inflammatory cytokine and an anti-inflammatory myokine | F | GCTACCAAACTGGATATAATCAGGA | (2) |
|  |  | R | CCAGGTAGCTATGGTACTCCAGAA |  |
| IL10 | Interleukin 10  Anti-inflammatory cytokine | F | CAGAGCCACATGCTCCTAGA | (3) |
|  |  | R | TGTCCAGCTGGTCCTTTGTT |  |
| TNF-α | Tumour necrosis factor alpha  Pro-inflammatory cytokine | F | CTGTAGCCCACGTCGTAGC | (3) |
|  |  | R | TTGAGATCCATGCCGTTG |  |
| IFN-γ | Interferon gamma  Pro-inflammatory cytokine | F | TCTTCAGCAACAGCAAGGCG | (4) |
|  |  | R | GCGACTCCTTTTCCGCTTCC |  |
| Muc2 | Mucin 2  Protein coding gene for mucus formation | F | GCTGACGAGTGGTTGGTGAATG | (5) |
|  |  | R | GATGAGGTGGCAGACAGGAGAC |  |
| CLDN-1 | Tight junction protein claudin-1 | F | ACTCCTTGCTGAATCTGAACAGT | (6) |
|  |  | R | GGACACAAAGATTGCGATCAG |  |
| CLDN-2 | Tight junction protein claudin-2 | F | TGAACACGGACCACTGAAAG | (7) |
|  |  | R | TTAGCAGGAAGCTGGGTCAG |  |
| DEFA5 | Defensin Alpha 5  Antimicrobial and cytotoxic peptides | F | GGCCTCCAAAGGAGATAGACA | This study |
|  |  | R | CAGGCTGATCCTATCCACAAA |  |

**Table S4.** PERMANOVA statistics between microbiota sequence timepoints of beta diversity matrices for each treatment group. N=5/group.

| **NRBB01** | | | | | |
| --- | --- | --- | --- | --- | --- |
|  | Day -7 | Day 5 | Day 13 | Day 25 | Day 35 |
| Day -7 |  |  |  |  |  |
| Day 5 | 0.043 |  |  |  |  |
| Day 13 | 0.021 | 0.223 |  |  |  |
| Day 25 | 0.092 | 0.362 | 0.594 |  |  |
| Day 35 | 0.098 | 0.018 | 0.028 | 0.036 |  |
| **Mix** | | | | | |
|  | Day -7 | Day 5 | Day 13 | Day 25 | Day 35 |
| Day -7 |  |  |  |  |  |
| Day 5 | 0.024 |  |  |  |  |
| Day 13 | 0.02 | 0.927 |  |  |  |
| Day 25 | 0.015 | 0.665 | 0.412 |  |  |
| Day 35 | 0.046 | 0.041 | 0.072 | 0.063 |  |
| **PBS** | | | | | |
|  | Day -7 | Day 5 | Day 13 | Day 25 | Day 35 |
| Day -7 |  |  |  |  |  |
| Day 5 | 0.24 |  |  |  |  |
| Day 13 | 0.3 | 0.601 |  |  |  |
| Day 25 | 0.313 | 0.139 | 0.161 |  |  |
| Day 35 | 0.402 | 0.192 | 0.436 | 0.436 |  |
| **NRBB01 + ABX** | | | | | |
|  | Day -7 | Day 5 | Day 13 | Day 25 | Day 35 |
| Day -7 |  |  |  |  |  |
| Day 5 | 0.052 |  |  |  |  |
| Day 13 | 0.014 | 0.009 |  |  |  |
| Day 25 | 0.01 | 0.003 | 0.008 |  |  |
| Day 35 | 0.01 | 0.012 | 0.013 | 0.01 |  |
| **Mix + ABX** | | | | | |
|  | Day -7 | Day 5 | Day 13 | Day 25 | Day 35 |
| Day -7 |  |  |  |  |  |
| Day 5 | 0.098 |  |  |  |  |
| Day 13 | 0.105 | 0.009 |  |  |  |
| Day 25 | 0.009 | 0.007 | 0.01 |  |  |
| Day 35 | 0.016 | 0.011 | 0.013 | 0.007 |  |
| **PBS + ABX** | | | | | |
|  | Day -7 | Day 5 | Day 13 | Day 25 | Day 35 |
| Day -7 |  |  |  |  |  |
| Day 5 | 0.009 |  |  |  |  |
| Day 13 | 0.003 | 0.009 |  |  |  |
| Day 25 | 0.007 | 0.01 | 0.01 |  |  |
| Day 35 | 0.009 | 0.014 | 0.009 | 0.013 |  |

**Supplementary figures**


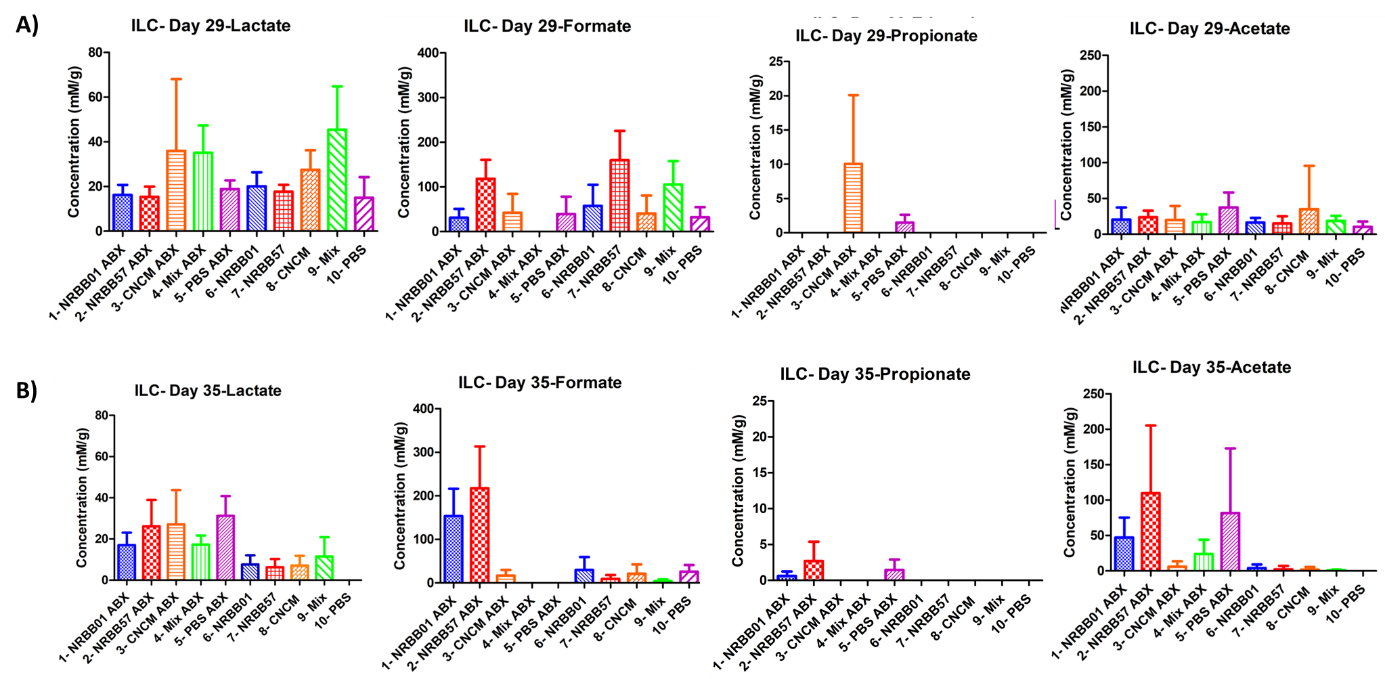


**Figure S1.** Ileum content HPLC VFA results for **A)** groups culled on Day 29, and **B)** Replicate groups culled on Day 35.


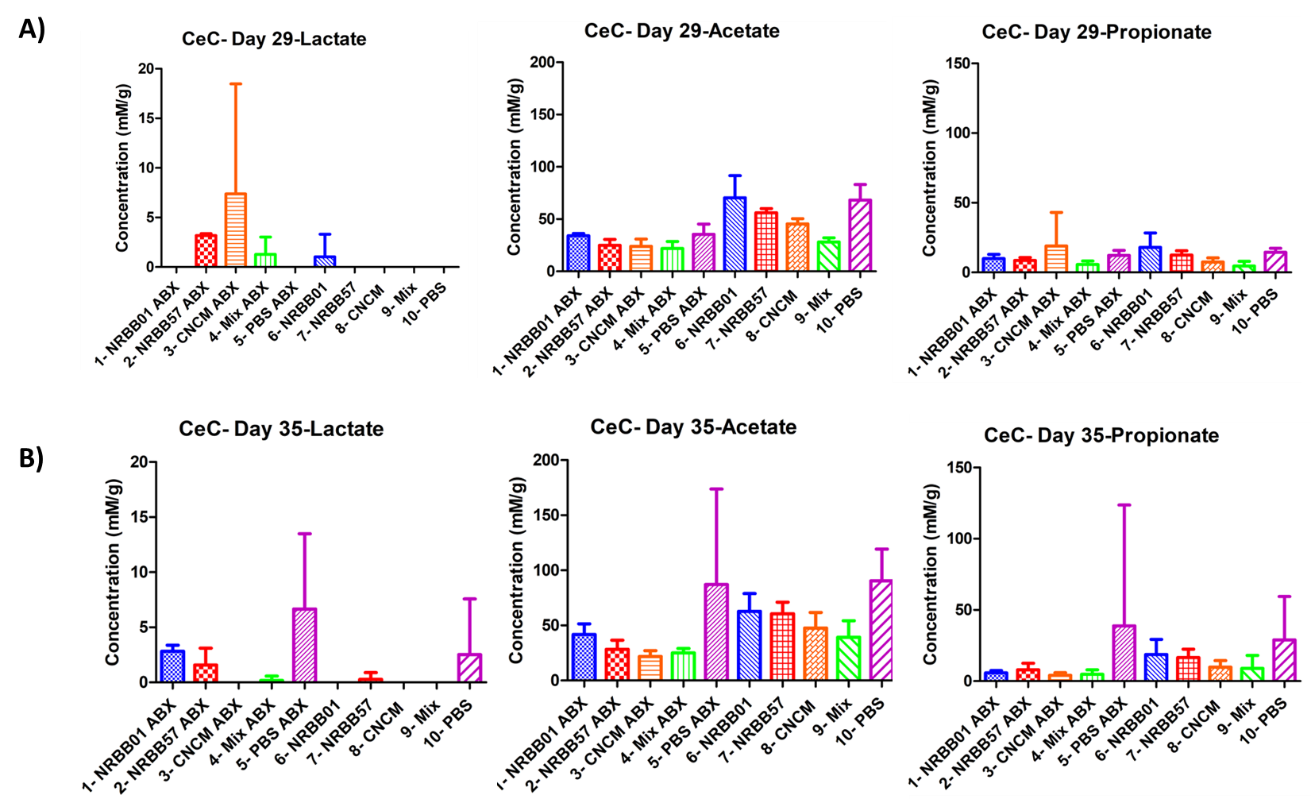
**Figure S2.** Cecum content HPLC VFA results for **A)** groups culled on Day 29, and **B)** replicate groups culled on Day 35.


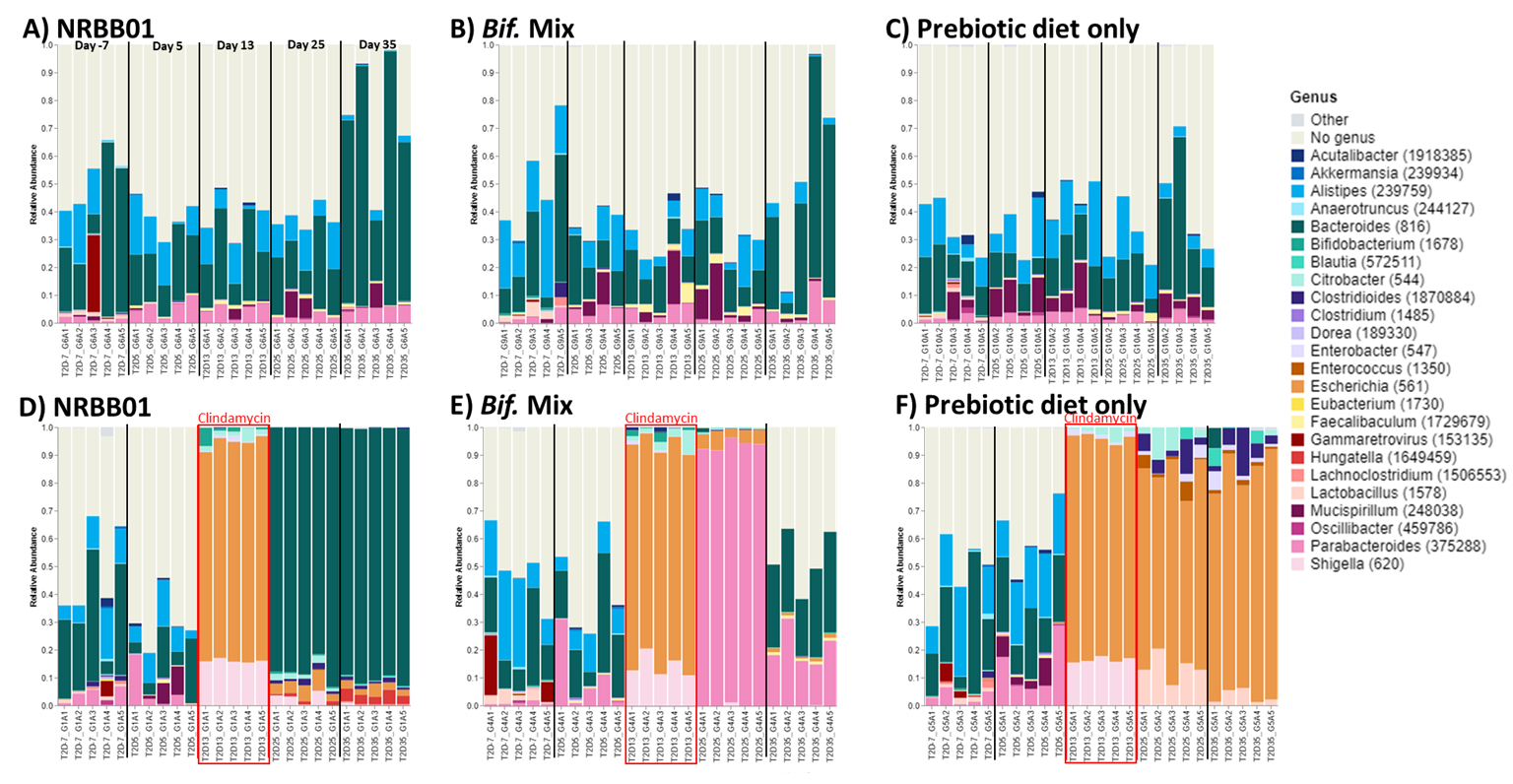


**Figure S3.** Relative abundance of the top 25 genera at Day -7, 5, 13, 25 and 35 across the 3 intervention groups supplemented with **A)** scGOS/lcFOS/2’-FL and *B. breve* NRBB01 **B)** scGOS/lcFOS/2’-FL and the *Bifidobacterium* strain Mix and **C)** scGOS/lcFOS/2’-FL diet only, without clindamycin treatment. Groups treated with clindamycin between Day 7-14 include groups supplemented with **D)** scGOS/lcFOS/2’-FL and B. breve NRBB01 **E)** scGOS/lcFOS/2’-FL and the *Bifidobacterium* strain Mix and **F)** scGOS/lcFOS/2’-FL diet only. N=5/group.


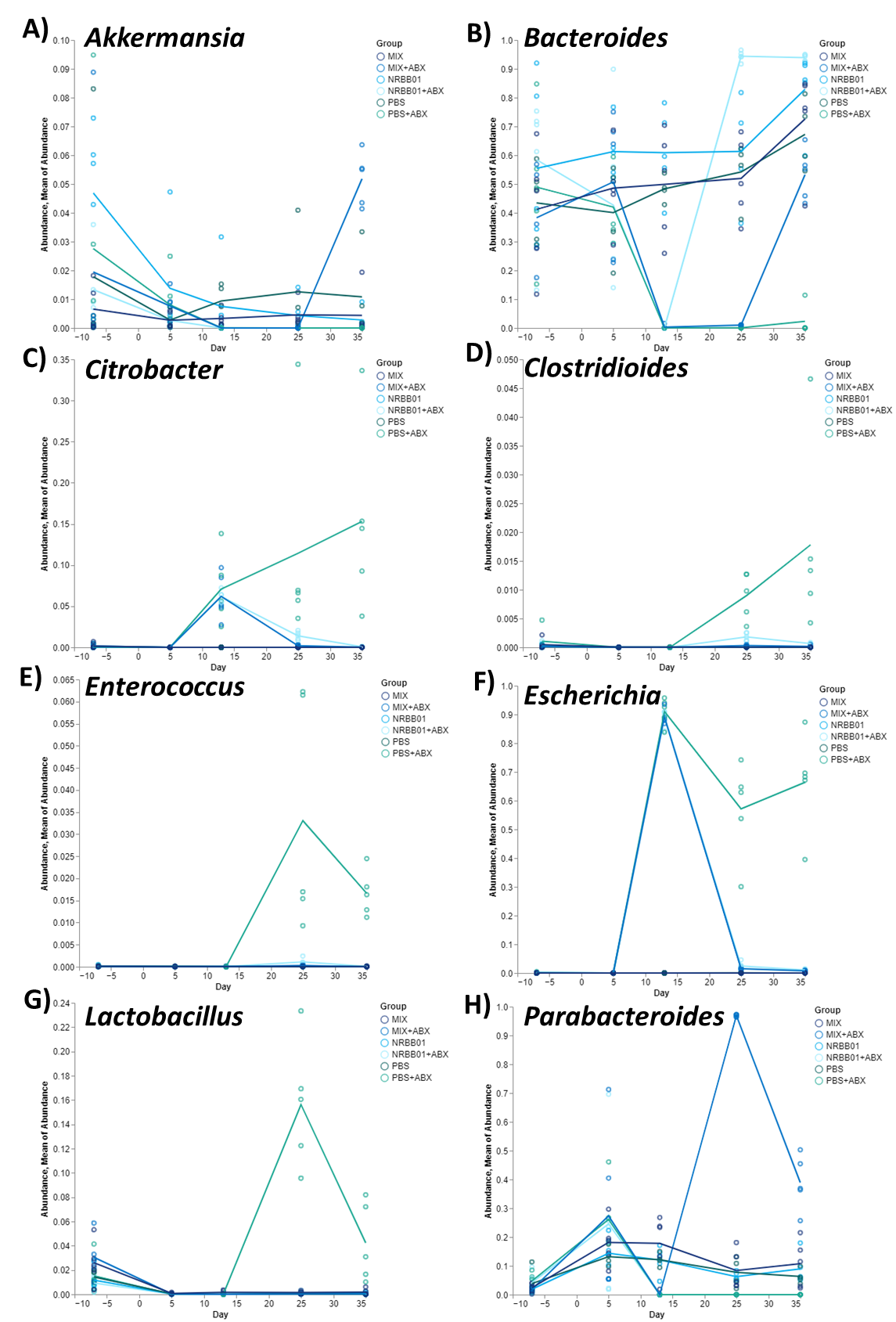


**Figure S4.** Line plot of the relative abundance of key genera **A)** *Akkermansia*, **B)** *Bacteroides*, **C)** *Citrobacter*, **D)** *Clostridioides*, **E)** *Enterococcus*, **F)** *Escherichia*, **G)** *Lactobacillus*, **H)** *Parabacteroides* at Days -7, 5, 13, 25 and 35. Each line represents a treatment group with and without antibiotic treatment (clindamycin) between Day 7-14 (*B. breve* NRBB01, *Bifidobacterium* strain Mix and scGOS/lcFOS/2’-FL diet only), without clindamycin treatment. Clindamycin treatment occurred between Day 7-14; n=5/group.


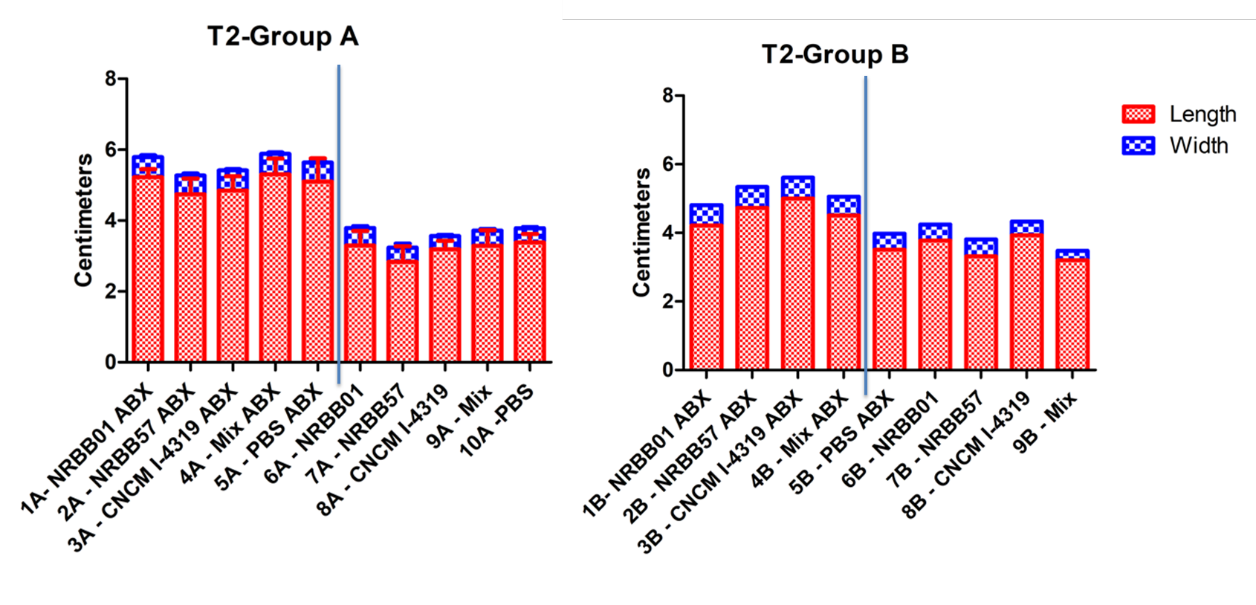


**Figure S5.** Cecum dimensions for replicate groups A (29 Day) and B (35 Day) demonstrate a difference between groups treated with and without antibiotics. Cecums of animals receiving clindamycin treatment are larger in length, which is more noticeable for Group A (cull Day 29) which was culled 2 weeks post antibiotic treatment (n = 5). Group B (cull Day 35) had an n=1 and PBS+ABX control was not imaged. Therefore, these results are only observational and not appropriate for statistical analysis.

**
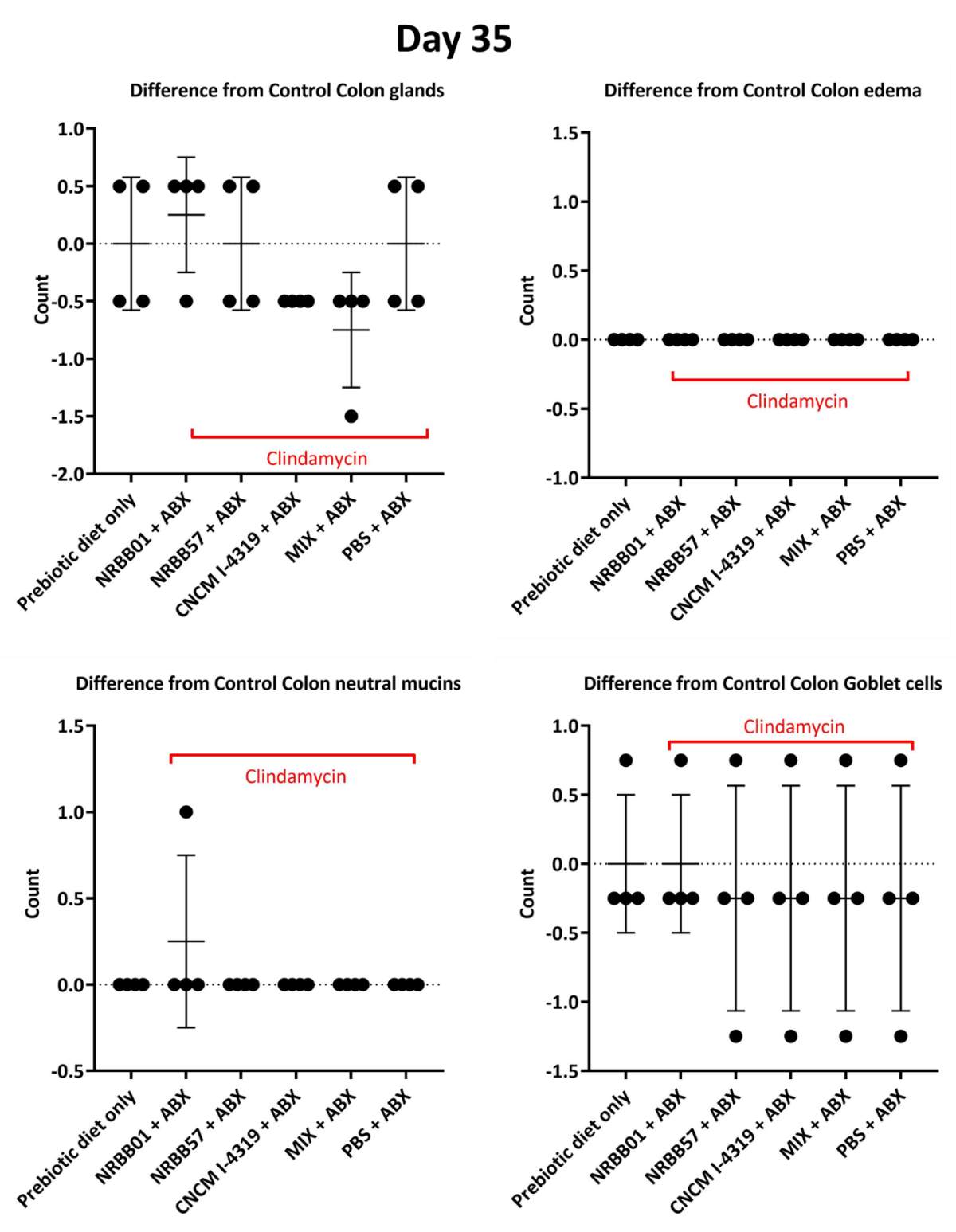
**

**Figure S6.** Histological scoring found no significant differences, at Day 35 (3 weeks post antibiotic), of the colonic folds number (glands), edema, neutral mucin staining, and goblets cells (I-L) were measured with at least 5 per mouse. Data are expressed as means ± SD. n= 4 per group. Mean values were significantly different between the groups: *P<0.05, **P<0.01, *** P<0.001.

**
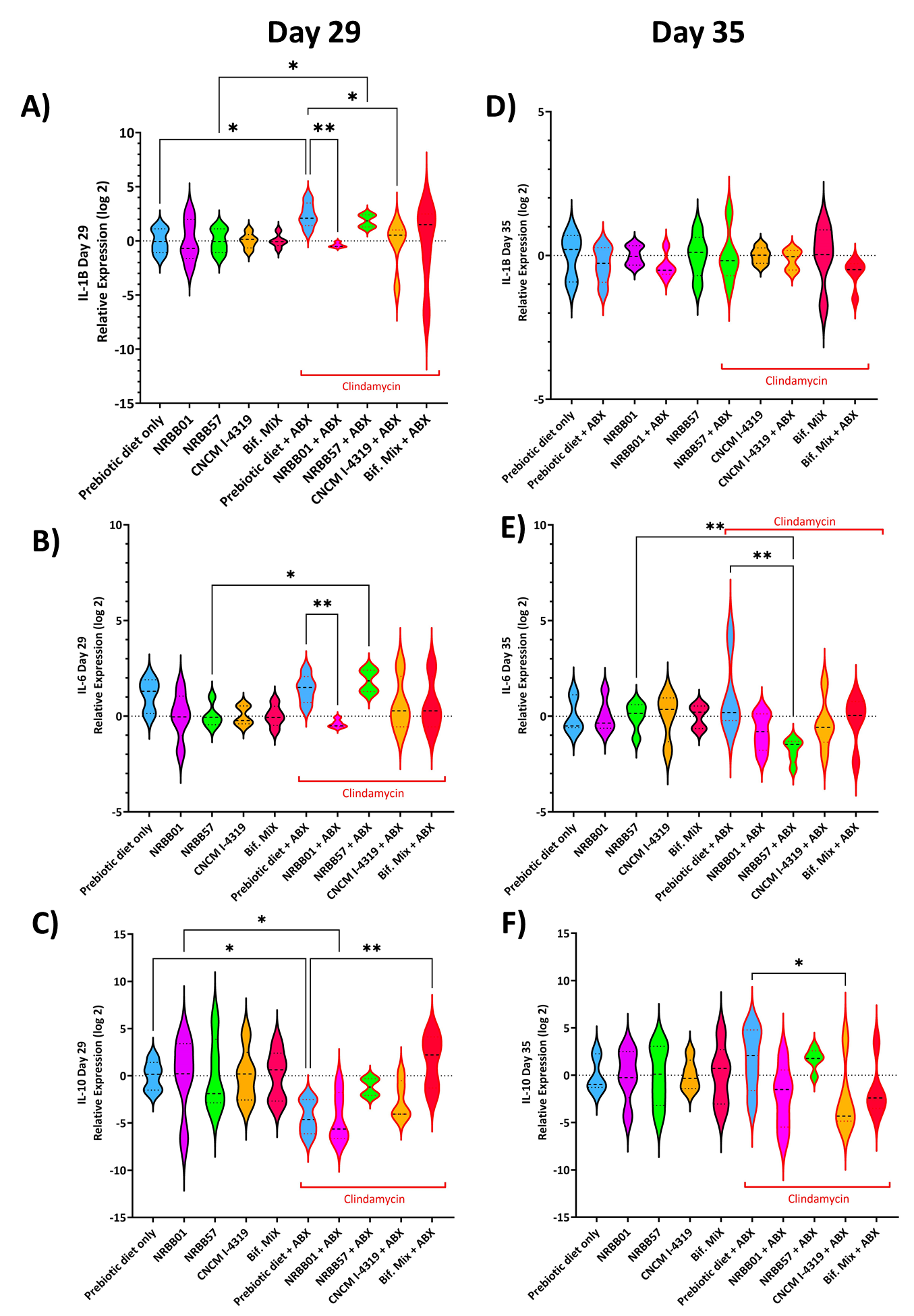
**

**Figure S7.** Immune markers **A)** IL-1β, **B)** lL-6, and **C)** IL-10 qPCR relative expressions at Day 29 and **D)** IL-1β, **E)** lL-6, and **F)** IL-10 at Day 35. Data are expressed as Log 2 transformed ± SD; n= 5 per group. Mean values were significantly different between the groups: *P<0.05, **P<0.01, *** P<0.001.

**
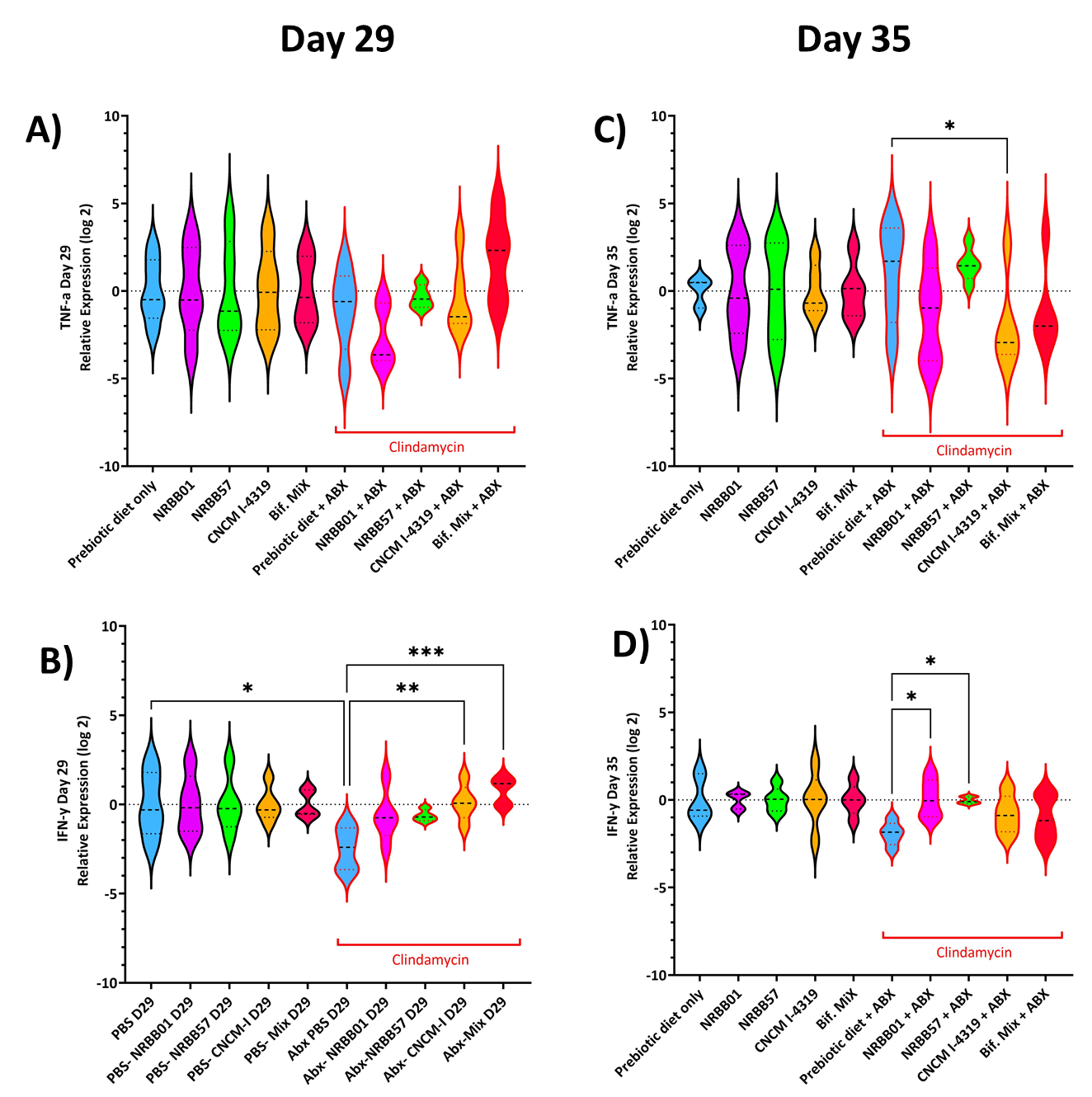
**

**Figure S8.** Immune markers **A)** TNF-α and **B)** IFN-γ qPCR relative expressions at Day 29 and **C)** TNF-α **D)** IFN-γ at Day 35. Data are Log 2 transformed ± SD; n= 5 per group. Mean values were significantly different between the groups: *P<0.05, **P<0.01, *** P<0.001.

**
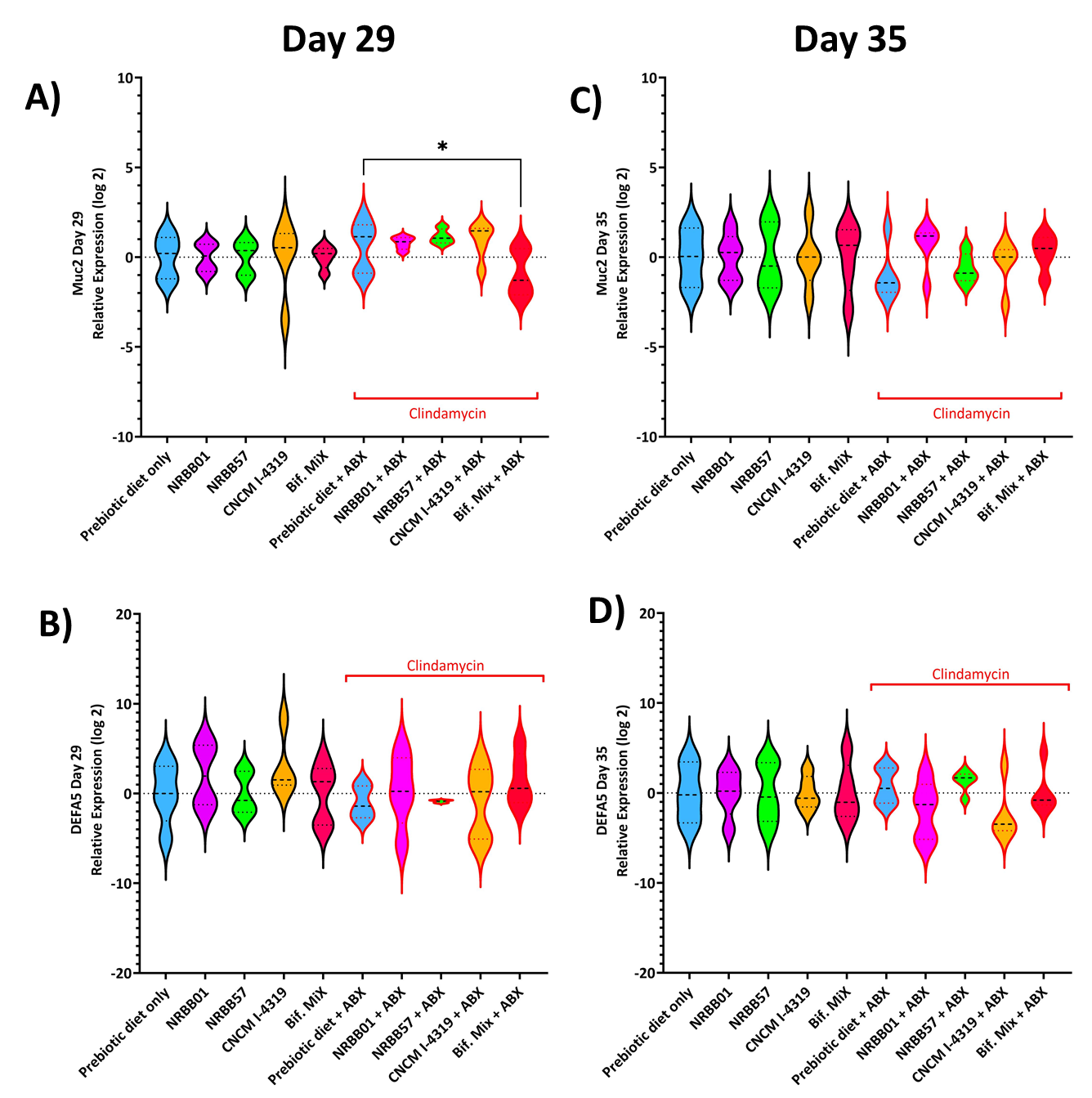
**

**Figure S9.** Epithelial barrier markers **A)** Muc2, and **B)** DEFA5 at Day 29 qPCR relative expressions and **C)** Muc2, **D)** DEFA5 at Day 35. Data is Log 2 transformed ± SD; n= 5 per group. Mean values were significantly different between the groups: *P<0.05, **P<0.01, *** P<0.001.

**
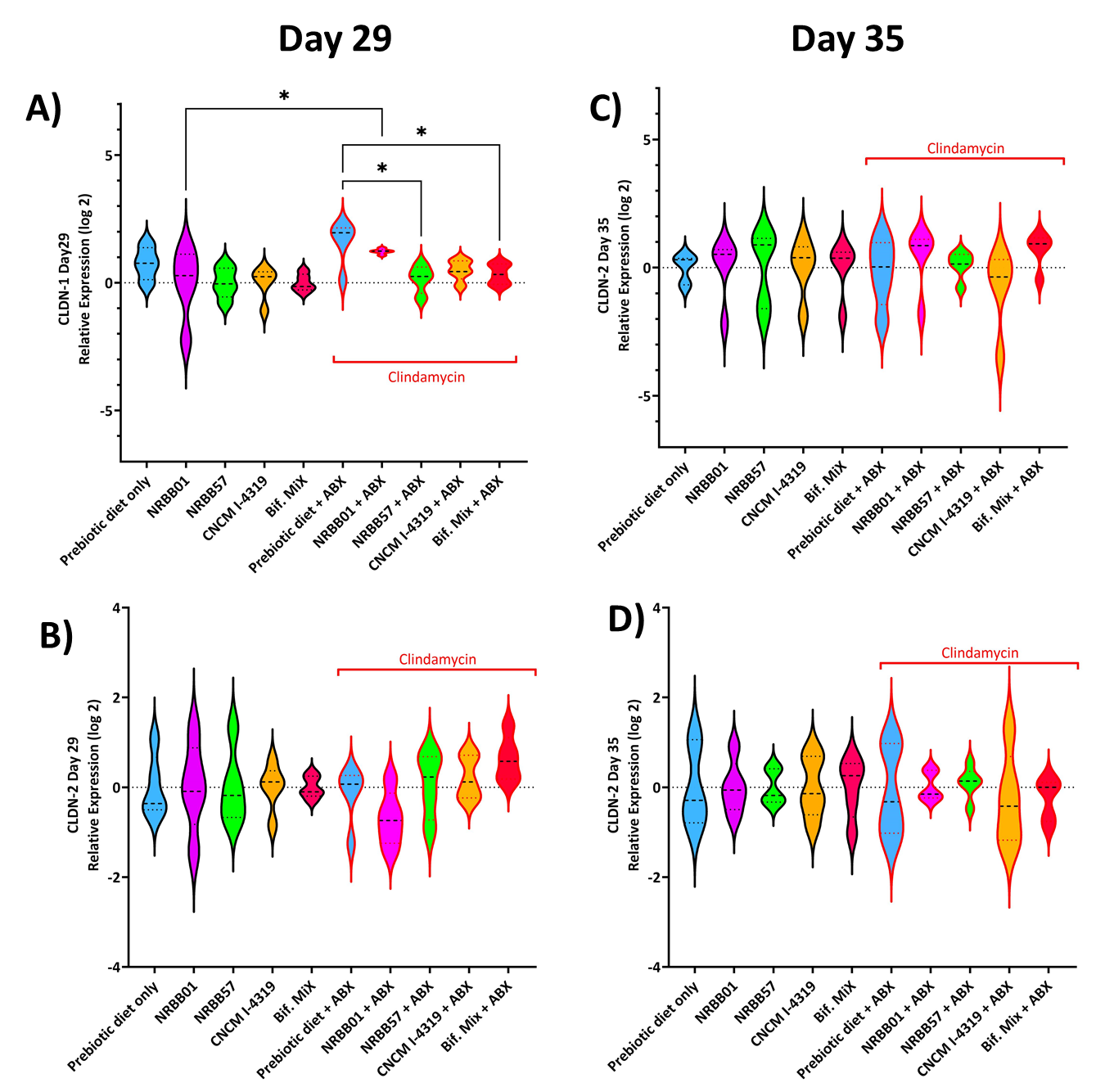
**

**Figure S10.** Epithelial barrier markers **A)** CLDN-1 and **A)** CLDN-2 qPCR relative expressions at Day 29 and **C)** CLDN-1, **D)** CLDN-2 at Day 35. Log 2 transformed data as ± SD; n= 5 per group. Mean values were significantly different between the groups: *P<0.05, **P<0.01, *** P<0.001.

**References**

1. F. Turroni *et al.*, Exploring the diversity of the bifidobacterial population in the human intestinal tract. *Appl Environ Microbiol* **75**, 1534-1545 (2009).

2. M. D. Thompson *et al.*, Enhanced Steatosis and Fibrosis in Liver of Adult Offspring Exposed to Maternal High-Fat Diet. *Gene Expr* **17**, 47-59 (2016).

3. N. Lubos *et al.*, Inflammation shapes pathogenesis of murine arrhythmogenic cardiomyopathy. *Basic Res Cardiol* **115**, 42-42 (2020).

4. I. Rostovsky, C. Davis, Induction of an Embryonic Mouse Innate Immune Response following Inoculation *In Utero* with Minute Virus of Mice. *Journal of Virology* **89**, 2182 (2015).

5. A.-L. Cattin *et al.*, Hepatocyte nuclear factor 4alpha, a key factor for homeostasis, cell architecture, and barrier function of the adult intestinal epithelium. *Mol Cell Biol* **29**, 6294-6308 (2009).

6. J. Falero-Perez, C. M. Sorenson, N. Sheibani, Retinal astrocytes transcriptome reveals Cyp1b1 regulates the expression of genes involved in cell adhesion and migration. *PLOS ONE* **15**, e0231752 (2020).

7. R. C. De Lisle, Disrupted tight junctions in the small intestine of cystic fibrosis mice. *Cell Tissue Res* **355**, 131-142 (2014).
